# Ovarian cancer screening and mortality in the UK Collaborative Trial of Ovarian Cancer Screening (UKCTOCS): a randomised controlled trial

**DOI:** 10.1016/S0140-6736(15)01224-6

**Published:** 2016-03-05

**Authors:** Ian J Jacobs, Usha Menon, Andy Ryan, Aleksandra Gentry-Maharaj, Matthew Burnell, Jatinderpal K Kalsi, Nazar N Amso, Sophia Apostolidou, Elizabeth Benjamin, Derek Cruickshank, Danielle N Crump, Susan K Davies, Anne Dawnay, Stephen Dobbs, Gwendolen Fletcher, Jeremy Ford, Keith Godfrey, Richard Gunu, Mariam Habib, Rachel Hallett, Jonathan Herod, Howard Jenkins, Chloe Karpinskyj, Simon Leeson, Sara J Lewis, William R Liston, Alberto Lopes, Tim Mould, John Murdoch, David Oram, Dustin J Rabideau, Karina Reynolds, Ian Scott, Mourad W Seif, Aarti Sharma, Naveena Singh, Julie Taylor, Fiona Warburton, Martin Widschwendter, Karin Williamson, Robert Woolas, Lesley Fallowfield, Alistair J McGuire, Stuart Campbell, Mahesh Parmar, Steven J Skates

**Affiliations:** a Department of Women's Cancer, Institute for Women's Health, University College London, London, UK; b University of New South Wales, Sydney, NSW, Australia; c Obstetrics and Gynaecology, School of Medicine, College of Biomedical and Life Sciences, Cardiff University, Cardiff, UK; d Research Department of Pathology, Cancer Institute, University College London Hospital, London, UK; e Clinical Biochemistry, University College London Hospital, London, UK; f Department of Gynaecological Oncology, University College London Hospital, London, UK; g Department of Gynaecological Oncology, James Cook University Hospital, Middlesbrough, UK; h Department of Gynaecological Oncology, Belfast City Hospital, Belfast, UK; i Malomatia (Information, Communication and Technology QATAR) Qatari Shareholding Company, Qatar; j Northern Gynaecological Oncology Centre, Queen Elizabeth Hospital, Gateshead, Tyne and Wear, UK; k Medical Research Council Centre for Neuromuscular Diseases, National Hospital for Neurology and Neurosurgery, London, UK; l School of Medical Sciences, Bangor University, Bangor, Gwynedd, UK; m Department of Gynaecology, Liverpool Women's Hospital, Liverpool, UK; n Women's Hospital, Hamad Medical Corporation, Doha, Qatar; o Department of Gynaecological Oncology, Royal Derby Hospital, Derby, UK; p Department of Gynaecological Oncology, Llandudno Hospital, Gwynedd, UK; q Derriford Hospital, Plymouth, Devon, UK; r Department of Gynaecological Oncology, Royal Free Hospital, London; s Department of Gynaecological Oncology, St Michael's Hospital, Bristol, UK; t Department of Gynaecological Oncology, St Bartholomew's Hospital, London, UK; u Biostatistics Center, Massachusetts General Hospital, Boston, MA, USA; v Central Manchester Foundation Trust, St Mary's Hospital, Manchester, UK; w Institute of Cancer Sciences, University of Manchester, Manchester, UK; x Department of Gynaecological Oncology, University Hospital of Wales, Heath Park, Cardiff, UK; y Department of Pathology, Barts Health National Health Service Trust, London, UK; z Public Health England, London, UK; aa Department of Gynaecological Oncology, Nottingham City Hospital, Nottingham, UK; ab Department of Gynaecological Oncology, Queen Alexandra Hospital, Portsmouth, Hampshire, UK; ac Sussex Health Outcomes Research and Education in Cancer, Brighton and Sussex Medical School, University of Sussex, Sussex, UK; ad Department of Social Policy, London School of Economics, London, UK; ae Create Health Clinic, London, UK; af Medical Research Council Clinical Trials Unit at University College London, London, UK; ag Harvard Medical School, Boston, MA, USA; ah Centre for Women's Health, Institute of Human Development, University of Manchester, Manchester, UK

## Abstract

**Background:**

Ovarian cancer has a poor prognosis, with just 40% of patients surviving 5 years. We designed this trial to establish the effect of early detection by screening on ovarian cancer mortality.

**Methods:**

In this randomised controlled trial, we recruited postmenopausal women aged 50–74 years from 13 centres in National Health Service Trusts in England, Wales, and Northern Ireland. Exclusion criteria were previous bilateral oophorectomy or ovarian malignancy, increased risk of familial ovarian cancer, and active non-ovarian malignancy. The trial management system confirmed eligibility and randomly allocated participants in blocks of 32 using computer-generated random numbers to annual multimodal screening (MMS) with serum CA125 interpreted with use of the risk of ovarian cancer algorithm, annual transvaginal ultrasound screening (USS), or no screening, in a 1:1:2 ratio. The primary outcome was death due to ovarian cancer by Dec 31, 2014, comparing MMS and USS separately with no screening, ascertained by an outcomes committee masked to randomisation group. All analyses were by modified intention to screen, excluding the small number of women we discovered after randomisation to have a bilateral oophorectomy, have ovarian cancer, or had exited the registry before recruitment. Investigators and participants were aware of screening type. This trial is registered with ClinicalTrials.gov, number NCT00058032.

**Findings:**

Between June 1, 2001, and Oct 21, 2005, we randomly allocated 202 638 women: 50 640 (25·0%) to MMS, 50 639 (25·0%) to USS, and 101 359 (50·0%) to no screening. 202 546 (>99·9%) women were eligible for analysis: 50 624 (>99·9%) women in the MMS group, 50 623 (>99·9%) in the USS group, and 101 299 (>99·9%) in the no screening group. Screening ended on Dec 31, 2011, and included 345 570 MMS and 327 775 USS annual screening episodes. At a median follow-up of 11·1 years (IQR 10·0–12·0), we diagnosed ovarian cancer in 1282 (0·6%) women: 338 (0·7%) in the MMS group, 314 (0·6%) in the USS group, and 630 (0·6%) in the no screening group. Of these women, 148 (0·29%) women in the MMS group, 154 (0·30%) in the USS group, and 347 (0·34%) in the no screening group had died of ovarian cancer. The primary analysis using a Cox proportional hazards model gave a mortality reduction over years 0–14 of 15% (95% CI −3 to 30; p=0·10) with MMS and 11% (−7 to 27; p=0·21) with USS. The Royston-Parmar flexible parametric model showed that in the MMS group, this mortality effect was made up of 8% (−20 to 31) in years 0–7 and 23% (1–46) in years 7–14, and in the USS group, of 2% (−27 to 26) in years 0–7 and 21% (−2 to 42) in years 7–14. A prespecified analysis of death from ovarian cancer of MMS versus no screening with exclusion of prevalent cases showed significantly different death rates (p=0·021), with an overall average mortality reduction of 20% (−2 to 40) and a reduction of 8% (−27 to 43) in years 0–7 and 28% (−3 to 49) in years 7–14 in favour of MMS.

**Interpretation:**

Although the mortality reduction was not significant in the primary analysis, we noted a significant mortality reduction with MMS when prevalent cases were excluded. We noted encouraging evidence of a mortality reduction in years 7–14, but further follow-up is needed before firm conclusions can be reached on the efficacy and cost-effectiveness of ovarian cancer screening.

**Funding:**

Medical Research Council, Cancer Research UK, Department of Health, The Eve Appeal.

## Introduction

The poor prognosis for ovarian cancer^1^ motivated us to start a programme of screening research 30 years ago.^2^ We have since reported CA125 as a predictor of ovarian cancer risk,^3^, ^4^ high specificity^2^ and preliminary evidence of a survival benefit^5^ of multimodal screening using CA125 interpreted with a cutoff with transvaginal ultrasound as a second-line test, development of a risk of ovarian cancer algorithm (ROCA) for interpretation of longitudinal CA125,^6^, ^7^ use of morphological criteria in second-line transvaginal ultrasound,^8^ and use of ROCA in a pilot randomised controlled trial.^9^ During this period, advances in treatment have only produced a slight reduction in disease mortality.^10^, ^11^

**Research in context**

**Evidence before this study**

During the 1990s, findings from large prospective studies of screening showed that both CA125 and ultrasound-based ovarian cancer screening could identify preclinical cases of ovarian cancer. These studies included some by our own group with use of a multimodal strategy incorporating CA125 with ultrasound as a secondary test. In the year before the start of this trial, a systematic review of 25 ovarian cancer screening studies commissioned by the National Health Service Health Technology Assessment Programme reported that although ultrasound and multimodal screening can detect ovarian cancer in asymptomatic women, the effect of screening on ovarian cancer was unproven. The authors concluded that screening should not be introduced into clinical practice until further information was available from randomised trials designed to assess the effect of ovarian cancer screening on mortality and its adverse effects and cost-effectiveness. After that publication, our group reported the findings of a pilot randomised controlled trial in 22 000 postmenopausal women showing the feasibility of ovarian cancer screening with use of a multimodal screening strategy and provided preliminary evidence of a survival benefit in this population. During this trial, two large randomised controlled trials were done: the Shizuoka Cohort Study of Ovarian Cancer Screening and the ovarian component of the Prostate Lung Colorectal Ovarian (PLCO) Cancer Screening Trial. The Shizuoka Cohort Study of Ovarian Cancer Screening only reported on ovarian cancer detection and not on deaths. Findings from the ovarian component of the PLCO Cancer Screening Trial showed no difference in ovarian cancer deaths between the screening and control groups. We searched MEDLINE between Jan 1, 2001, and Nov 31, 2015, using the protocol described by Mosch and colleagues, with the following search terms: “ovarian neoplasms”, “Fallopian tube neoplasms”, “ovar*”, “fallopian tub* OR adnex*”, “tumo*”, “malignan*”, “carcinoma* OR adenocarcinoma* OR neoplasm* OR mass*”, “mass screening”, “early detection of cancer”, “randomized controlled trial”, “controlled clinical trial”, “randomized”, “placebo”, “clinical trials”, “randomly”, and “trial”. This search yielded 234 publications, which, when limited to randomised controlled trials of human female adults published in the English language resulted in 64 articles, 11 of which were duplicated. The remaining 53 articles consisted of 28 pertaining to the PLCO Cancer Screening Trial, 11 from our own group, and one from Kobayashi and colleagues in 2008 (the Shizuoka Cohort Study of Ovarian Cancer Screening). We identified three key randomised controlled trials in ovarian cancer screening. Of these, only the PLCO Cancer Screening Trial thus far has reported mortality data.

**Added value of this study**

To our knowledge, this trial is the first randomised controlled trial of ovarian cancer screening to produce findings that show that in postmenopausal women from the general population, annual screening with use of the multimodal strategy is safe and could reduce deaths due to ovarian cancer. These findings are derived from one of the largest randomised trials ever done and renew hope that death rates from the most lethal of all gynaecological malignancies can be reduced through early detection.

**Implications of all the available evidence**

Our findings suggest that a multimodal approach to screening might detect ovarian cancer sufficiently early to reduce mortality. To establish the magnitude of this reduction in deaths, a longer duration of follow-up is needed. Meanwhile, efforts to refine ovarian cancer screening strategies should continue.

This research led us in 2001 to undertake a definitive randomised controlled trial, the UK Collaborative Trial of Ovarian Cancer Screening (UKCTOCS), of more than 200 000 women.^12^ The primary aim was to assess the effect of screening on disease mortality. We have previously reported on performance characteristics of screening,^13^, ^14^ harms related to false-positive surgery,^13^, ^14^ and the psychological morbidity associated with screening.^15^ This report is a key landmark for the programme, providing the first ovarian cancer mortality data from UKCTOCS.

## Methods

### Study design and participants

In this randomised controlled trial, we recruited women through 13 regional centres (RCs) in National Health Service (NHS) Trusts in England, Wales, and Northern Ireland, with the Queen Mary University of London as the coordinating centre (CC) between 2001 and 2004 and then University College London from 2004 onwards. We invited women identified through the age-sex registers of 27 participating primary care trusts within the RC catchment areas. We commissioned specialised software from the NHS to randomly select women aged 50–74 years and then flag them on primary care trusts' registers and allow electronic transfer of their personal and general practice details to the CC in lots of 5000 to 10 000 every quarter. We then sent women personal invitations and logged replies on the trial management system. Women attended a recruitment clinic at the RC where they viewed an information video, completed a recruitment questionnaire, and provided written consent and a baseline serum sample. We scanned recruitment questionnaires at the CC into a bespoke trial management system.^12^ Eligibility criteria were 50–74 years of age and postmenopausal status. Exclusion criteria were self-reported previous bilateral oophorectomy or ovarian malignancy, increased risk of familial ovarian cancer, or active non-ovarian malignancy.

The trial design has been previously published^12^, ^13^, ^14^ and the protocol is available online. Ethical approval was by the UK North West Multicentre Research Ethics Committees (North West MREC 00/8/34).

### Randomisation and masking

The trial management system confirmed eligibility and then randomly allocated women to annual screening using a multimodal screening (MMS) or ultrasound screening (USS) strategy or no screening in a 1:1:2 ratio. The randomisation was accomplished using the Visual Basic randomisation statement and the Rnd function. The trial management system allocated a set of 32 random numbers to each RC, of which eight were allocated to MMS, eight to USS, and the remaining 16 to no screening. We randomly allocated each successive volunteer within the RC to one of the numbers and subsequently randomly allocated them into a group. Investigators and participants were aware of screening type, but the outcomes committee was masked.

### Procedures

Annual screening in the MMS group used serum CA125 concentration testing, with the pattern over time interpreted with use of the risk of ovarian cancer calculation, which identifies significant rises in CA125 concentration above baseline.^6^, ^7^, ^9^ Next, ROCA triaged women to normal (annual screening), intermediate (repeat CA125 concentration testing in 3 months), and elevated (repeat CA125 concentration testing and transvaginal USS as a second-line test in 6 weeks) risk. Annual screening in the USS group used transvaginal USS as the primary test, which was classified as normal (annual screening), unsatisfactory (repeat in 3 months), or abnormal (scan with a senior ultrasonographer within 6 weeks).^13^ In both groups, women with persistent abnormalities had clinical assessment and additional investigations within the NHS by a trial clinician.^13^, ^14^ We deemed women who had surgery or a biopsy for suspected ovarian cancer after clinical assessment screen positive.^13^, ^14^

Screening was implemented centrally by the CC using a web-based trial management system, which ensured that protocol deviations were kept to a minimum.^12^ A quality assurance programme for transvaginal USS and accreditation for scanning of postmenopausal ovaries was overseen by the ultrasound committee.^16^ We linked women using their NHS number in England and Wales to the Health and Social Care Information Centre for cancer and death registrations and, in Northern Ireland, to the Central Services Agency and Northern Ireland Cancer Registry. For women from English RCs, we also obtained data for cancer diagnosis from the National Cancer Intelligence Network, and between April, 2001, and March, 2010, Hospital Episodes Statistics administrative records. Other sources were two postal follow-up questionnaires (3–5 years after randomisation and April, 2014) and direct communication from trial participants, their families, and physicians.

We interrogated all available data sources to identify women diagnosed after randomisation with any of 19 International Classification of Diseases (ICD)-10 codes (appendix p 3).^14^ We retrieved copies of medical notes for all except those with ICD-10 C80 (malignant neoplasm of uncertain origin) who also had another specific non-ovarian or non-peritoneal cancer registration (appendix p 25). All were reviewed by an outcomes review committee (two pathologists and two gynaecological oncologists) who were masked to the randomisation group. We used an algorithm to assign the final diagnosis. We based death due to ovarian cancer on disease progression (appearance of new lesions or increases in size of previously documented lesions with imaging, clinical worsening, or rising biomarker concentrations). We ascertained ovarian and adnexal surgery outside of the trial after randomisation through both self-reporting and Hospital Episodes Statistics records.

We also ascertained contamination in the no screening group. This contamination was based on self-reporting in the 2014 follow-up questionnaire in which women in the no screening group were asked whether they had had ovarian cancer screening after recruitment.

The original trial protocol specified six annual screens and follow-up for 7 years from randomisation.^12^ In 2008, an analysis of overall and cause-specific standardised mortality in the no screening group showed a lower than expected mortality rate. We therefore extended screening in the USS and MMS groups to Dec 31, 2011, resulting in women being offered 7–11 screens depending on the year of randomisation. Follow-up was extended to Dec 31, 2014.

### Outcomes

The primary outcome was ovarian cancer death by Dec 31, 2014, assessed with use of data from the sources described above. Ovarian cancer refers to malignant neoplasms of the ovary (ICD-10 C56), which include primary non-epithelial ovarian cancer, borderline epithelial ovarian cancer, and invasive epithelial ovarian cancer; malignant neoplasms of the fallopian tube (ICD-10 C57.0); and undesignated malignancies of the ovaries, fallopian tube, or peritoneum.^17^ It does not include primary peritoneal cancer, which was diagnosed on the basis of WHO 2003 criteria.^18^

A secondary outcome was death due to ovarian and primary peritoneal cancer (ICD-10 C48.1 and C48.2). Most peritoneal cancers are likely to be classified as tubal and ovarian cancer once wider acceptance of the WHO 2014 revision has occurred.^19^ Another secondary outcome was compliance with screening, which was the proportion of women who attended all tests that consisted of a screening episode out of the total who were eligible for that screening episode. A further secondary outcome was complications related to screening and false-positive surgery. Centres reported screening-related complications to a designated safety officer. We deemed screen-positive surgery resulting in benign pathology or normal adnexa false positive. Medical notes were centrally reviewed for complications, which were classified as major (resulting in sequelae) or minor by a designated gynaecological oncologist. Other secondary outcomes were to assess and compare the performance characteristics of the two screening strategies, assess the psychological effects of screening, and establish the resource implications of screening.

### Statistical analysis

In 2000, we estimated that a sample size of 200 000 women at a two-sided 5% significance level for a difference in relative ovarian cancer mortality of 30% would give 90% power for the no screening versus combined screening groups and 80% power for the no screening versus MMS or no screening versus USS comparisons. After trial extension to Dec 31, 2014, our recalculated power remained 80% at a two-sided 5% significance level to detect a reduction of 30% in no screening versus MMS or no screening versus USS comparisons.

The primary mortality analysis was an MMS versus no screening and USS versus no screening analysis of the primary outcome with use of a Cox proportional hazards model. The primary analysis initially consisted of comparison of the combined MMS and USS with the no screening group and individual comparisons of MMS with no screening and USS with no screening. The assumption was that sensitivity of the two screening strategies would be similar. During the trial, the data monitoring and ethics committee concluded that this assumption was not the case. On the basis of differences in performance characteristics of the two strategies at the initial screen,^13^ we updated our primary statistical analysis to MMS versus no screening and USS versus no screening comparisons. We defined survival time from date of randomisation to date of death due to ovarian cancer or censorship, or sooner if the volunteer died of another cause or was lost to follow-up. To allow for the fact that we were comparing two intervention arms against a control group, we made a Dunnett correction for multiple testing against a control to the critical α (α=0·0258). Mortality reduction estimates are 1 – hazard ratio (HR) estimates.

Early detection is likely to have less effect on women with ovarian cancer before screening starts (prevalent cases) than on those who develop it after the start of screening. The typical CA125 concentration profile noted before diagnosis in ovarian cancer cases is a baseline level, a change point, and then a rising CA125 concentration (appendix pp 10, 25).^14^ We did a prespecified MMS versus no screening subgroup analysis excluding prevalent cases for which the estimated change point was before randomisation, showing that the cancer was present before screening began (appendix pp 10, 24). For the prespecified MMS versus no screening and USS versus no screening secondary outcome analysis of ovarian and peritoneal cancer deaths, we used a Cox proportional hazards model, Royston-Parmar (RP) model, and post-hoc weighted log-rank (WLR) test.

In the original statistical plan, we did not make provision for the delayed effect on mortality that has been reported in other screening trials.^20^, ^21^, ^22^, ^23^ The delayed effect is due to the inherent delay from randomisation to diagnosis and then death. Other cancer screening trials, including the Prostate Lung Colorectal Ovarian (PLCO) Cancer Screening Trial^24^, ^25^, ^26^, ^27^ and the National Lung Screening Trial,^28^ used a WLR test for the primary analysis to address this delay.^29^, ^30^ In view of the precedent of the PLCO Cancer Screening Trial screening trial, we did a sole post-hoc WLR analysis for the primary outcome, applying the WLR test with the same choice of weights proportional to pooled ovarian cancer mortality (appendix p 11) as that used in the primary PLCO Cancer Screening Trial analysis.^24^ We applied the prespecified RP method^31^ that can model proportional and non-proportional hazards due to delayed effects^22^, ^23^ to investigate the hazard functions for each group. We estimated changing HRs across time^32^ and calculated average HRs for a split midway between the first (0–7) and second (7–14) 7 years to summarise delayed effects.

We calculated the preliminary number needed to screen to prevent one death from ovarian cancer as the reciprocal of the absolute difference in cumulative mortality from ovarian cancer between the MMS and no screening groups. We calculated prespecified ovarian cancer survival in the no screening group from date of diagnosis to explore external validity by comparison with UK ovarian cancer survival statistics. In addition to non-parametric Kaplan-Meier plots, we investigated the underlying ovarian cancer incidence (hazard) rates for each group with RP models.

We did sensitivity analyses using both a prespecified Cox proportional hazards model and the post-hoc WLR to establish the robustness of the primary analysis. We did a data source analysis restricting the outcome to events that had a national cancer registration or death certification to address the possibility of under-reporting in the no screening group. We also did potential within-centre correlation analyses by stratifying the model by centre and using cluster-robust SEs. Finally, we did competing-risk analysis that treats other deaths as a competing risk rather than a censoring event. We also treated bilateral salpingo-oophorectomy done in women both within and outside of the trial as a competing (risk) event.^33^

As detailed previously,^13^, ^14^ we changed the risk of ovarian cancer calculation cutoffs in the MMS group in April, 2005, to maintain annual triage rates of 15% of women to intermediate-risk and 2% to increased-risk categories. Safety data related to screening was monitored annually by the data monitoring and ethics committee, who also assessed performance characteristics of the screening strategies. We did an interim analysis of the primary outcome when about half of the anticipated number of ovarian cancer deaths had occurred in the no screening group. We had no stopping guidelines for futility, and the critical significance level guideline for stopping for benefit was small (significance level of 0·001).

All analyses were by modified intention to screen. We analysed all randomly allocated women except for those who we later came to know had a bilateral oophorectomy, ovarian cancer, or exited the registry before recruitment. We did all statistical analyses using Stata (version 14), R (version 3.2.1; packages flexsurv and PwrGSD), and Stan (version 2.8.0).^34^

This trial is registered with ClinicalTrials.gov, number NCT00058032.

### Role of the funding source

The funders of the study had no role in study design, data collection, data analysis, data interpretation, or writing of the report. UM and AR extracted the dataset. MB, SJS, DJR, UM, and AR had full access to all the data in the study. IJJ, UM, SJS, and MP had final responsibility for the decision to submit for publication.

## Results

**Figure 1:**
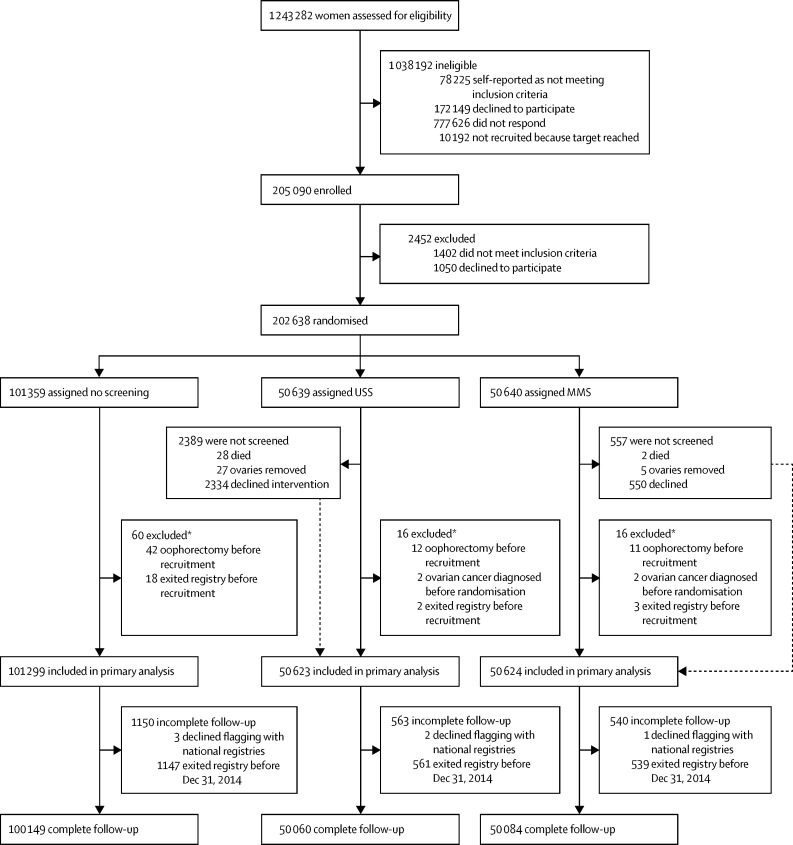
Trial profile MMS=multimodal screening. USS=ultrasound screening. *Events occurred before recruitment, but discovered after randomisation.

We invited 1 243 282 women to participate, recruiting 205 090 between April 17, 2001, and Sept 29, 2005, randomising 202 638 (16·3%) between June 1, 2001, and Oct 21, 2005: 101 359 (50·0%) to no screening, 50 640 (25·0%) to MMS, and 50 639 (25·0%) to USS. By end of screening on Dec 31, 2011, women attended 345 570 MMS and 327 775 USS annual screening episodes. We excluded 92 (<0·5%) women (no screening 60 [<0·5%]; MMS 16 [<0·5%]; USS 16 [<0·5%]) from the primary analysis (figure 1). The final cohort eligible for analysis consisted of 202 546 (>99·9%) women: 101 299 (>99·9%) in the no screening group, 50 624 (>99·9%) in the MMS group, and 50 623 (>99·9%) in the USS group. Baseline characteristics were balanced between study groups (table 1).

**Table 1:** Baseline characteristics

		**MMS (n=50 624)**	**USS (n=50 623)**	No screening (n=101 299)*	**Overall (n=202 546)**
Age at randomisation (years)		60·61 (56·03–66·15)	60·61 (55·99–66·15)	60·58 (55·97–66·15)	60·59 (55·99–66·15)
Time since last period at randomisation (years)		11·36 (5·26–18·49)	11·34 (5·24–18·46)	11·26 (5·22–18·46)	11·3 (5·23–18·47)
Duration of HRT use in those who were on HRT at randomisation (years)		8·09 (4·56–11·99)	8·15 (4·55–12·11)	8·17 (4·5–12·09)	8·15 (4·53–12·07)
Duration of OCP use in those who had used it (years)		5 (2–10)	5 (2–10)	5 (2–10)	5 (2–10)
Pregnancies <6 months		0 (0–1)	0 (0–1)	0 (0–1)	0 (0–1)
Children (pregnancies >6 months)		2 (2–3)	2 (2–3)	2 (2–3)	2 (2–3)
Height (cm)		162·6 (157·5–165·1)	162·6 (157·5–165·1)	162·6 (157·5–165·1)	162·6 (157·5–165·1)
Weight (kg)		67·6 (60·3–76·2)	67·6 (60·3–76·2)	67·6 (60·3–76·2)	67·6 (60·3–76·2)
Ethnic origin					
	White	48 845 (96·5%)	48 749 (96·3%)	97 598 (96·3%)	195 192 (96·4%)
	Black	670 (1·3%)	717 (1·4%)	1377 (1·4%)	2764 (1·4%)
	Asian	442 (0·9%)	477 (0·9%)	936 (0·9%)	1855 (0·9%)
	Other	428 (0·8%)	424 (0·8%)	839 (0·8%)	1691 (0·8%)
	Missing	239 (0·5%)	256 (0·5%)	549 (0·5%)	1044 (0·5%)
Hysterectomy		9680 (19·1%)	9496 (18·8%)	18 990 (18·7%)	38 166 (18·8%)
Ever use of OCP		30 098 (59·5%)	30 308 (59·9%)	60 284 (59·5%)	120 690 (59·6%)
Use of HRT at recruitment		9457 (18·7%)	9383 (18·5%)	19 150 (18·9%)	37 990 (18·8%)
Personal history of cancer†		2973 (5·9%)	2974 (5·9%)	6105 (6·0%)	12 052 (6·0%)
Personal history of breast cancer		1848 (3·7%)	1891 (3·7%)	3912 (3·9%)	7651 (3·8%)
Maternal history of ovarian cancer		802 (1·6%)	778 (1·5%)	1579 (1·6%)	3159 (1·6%)
Maternal history of breast cancer		3159 (6·2%)	3206 (6·3%)	6619 (6·5%)	12 984 (6·4%)

Data are n (%) or median (IQR). MMS=multimodal screening. USS=ultrasound screening. HRT=hormone replacement therapy. OCP=oral contraceptive pill.

* One woman asked for all her details to be removed.

† Includes those with personal history of breast cancer.

The last notification from the Health and Social Care Information Centre was received on March 25, 2015, and from Northern Ireland, for deaths, on April 9, 2015, and for cancer, on April 15, 2015. Complete follow-up until study completion (Dec 31, 2014) or death was possible in 200 293 (98·9%) women (no screening 100 149 [98·8%]; MMS 50 084 [98·9%]; USS 50 060 [98·9%]). Median follow-up time was 11·1 years (IQR 10·0–12·0) for all groups.

Of 3110 women investigated (appendix p 4), 1282 (41%) women were confirmed on outcomes review to have ovarian cancer (table 2). The overall sensitivity for detection of ovarian cancers, diagnosed within a year of a screening, was 84% (95% CI 79–88; 199 of 237) in the MMS group and 73% (66–79; 161 of 221) in the USS group. Of the primary peritoneal cancers, 81% (13 of 16) were screen detected with MMS and 30% (three of ten) were with USS. We noted evidence of a higher proportion of invasive epithelial ovarian and peritoneal cancer diagnosed with low-volume disease (stage I, II, and IIIa) in the MMS group (119 [40%] of 299; p<0·0001) than in the no screening group (149 [26%] of 574), but not in the USS group (62 [24%] of 259; p=0·57; appendix p 5).

**Table 2:** Ovarian and primary peritoneal cancers grouped by primary site and screening status

		Total*	**Screen positives**	**Cancers not detected by screening**			
				Screen negatives <1 year from last test of screening episode†	Screen negatives >1 year after last test of screening episode	After screening phase‡	Never attended screening
**MMS (50** **624 women, 548** **533 women-years)**							
Primary ovarian cancer		338 (100%)	199 (59%)	38 (11%)	41 (12%)	57 (17%)	3 (1%)
	Primary non-epithelial neoplasm of ovary (ICD C56)	11 (100%)	7 (64%)	2 (18%)	2 (18%)	0	0
	Primary borderline epithelial neoplasm of ovary (ICD C56)	44 (100%)	24 (55%)	10 (23%)	5 (11%)	5 (11%)	0
	Primary invasive epithelial neoplasm of ovary (ICD C56)	244 (100%)	147 (60%)	21 (9%)	29 (12%)	44 (18%)	3 (1%)
	Primary invasive epithelial neoplasm of fallopian tube (ICD C57.0)	19 (100%)	13 (68%)	2 (11%)	0	4 (21%)	0
	Undesignated (unable to delineate if primary site ovary or fallopian tube or peritoneum)	20 (100%)	8 (40%)	3 (15%)	5 (25%)	4 (20%)	0
Primary peritoneal cancer		16 (100%)	13 (81%)	3 (19%)	0	0	0
**USS (50** **623 women, 548** **825 women-years)**							
Primary ovarian cancer		314 (100%)	161 (51%)	60 (19%)	46 (15%)	34 (11%)	13 (4%)
	Primary non-epithelial neoplasm of ovary (ICD C56)	12 (100%)	11 (92%)	0	1 (8%)	0	0
	Primary borderline epithelial neoplasm of ovary (ICD C56)	53 (100%)	48 (91%)	2 (4%)	1 (2%)	0	2 (4%)
	Primary invasive epithelial neoplasm of ovary (ICD C56)	220 (100%)	93 (42%)	48 (22%)	37 (17%)	31 (14%)	11 (5%)
	Primary invasive epithelial neoplasm of fallopian tube (ICD C57.0)	13 (100%)	4 (31%)	3 (23%)	3 (23%)	3 (23%)	0
	Undesignated (unable to delineate if primary site ovary or fallopian tube or peritoneum)	16 (100%)	5 (31%)	7 (44%)	4 (25%)	0	0
Primary peritoneal cancer		10 (100%)	3 (30%)	3 (30%)	4 (40%)	0	0
**No screening (101** **299 women, 1** **097** **089 women-years)**							
Primary ovarian cancer		630 (100%)	..	501 (80%)	..	129 (20%)	..
	Primary non-epithelial neoplasm of ovary (ICD C56)	8 (100%)	..	7 (88%)	..	1 (13%)	..
	Primary borderline epithelial neoplasm of ovary (ICD C56)	62 (100%)	..	50 (81%)	..	12 (19%)	..
	Primary invasive epithelial neoplasm of ovary (ICD C56)	493 (100%)	..	392 (80%)	..	101 (20%)	..
	Primary invasive epithelial neoplasm of fallopian tube (ICD C57.0)	28 (100%)	..	21 (75%)	..	7 (25%)	..
	Undesignated (unable to delineate if primary site ovary or fallopian tube or peritoneum)	38 (100%)	..	30 (79%)	..	8 (21%)	..
	Primary ovarian neoplasm (histology not available)	1 (100%)	..	1 (100%)	..	0	..
Primary peritoneal cancer		15 (100%)	..	15 (100%)	..	0	..

Data are n (%). MMS=multimodal screening. ICD=International Classification of Diseases. USS=ultrasound screening.

* Includes in the MMS group, three screen-positive neoplasms of uncertain malignancy that have been recoded by outcomes review as non-epithelial ovarian cancer and one screen-positive borderline that has been recoded as invasive after the published incidence analysis; in the USS group, it includes five screen-positive neoplasms of uncertain malignancy that have been recoded by outcomes review as non-epithelial ovarian cancer and two additional screen-negative invasive epithelial ovarian cancers that were identified after the published prevalence analysis.

† For the no screening group, this category applies during the screening phase.

‡ Refers to more than 1 year after last potential screen in 2011 based on the anniversary of an individual's randomisation date.

**Figure 2:**
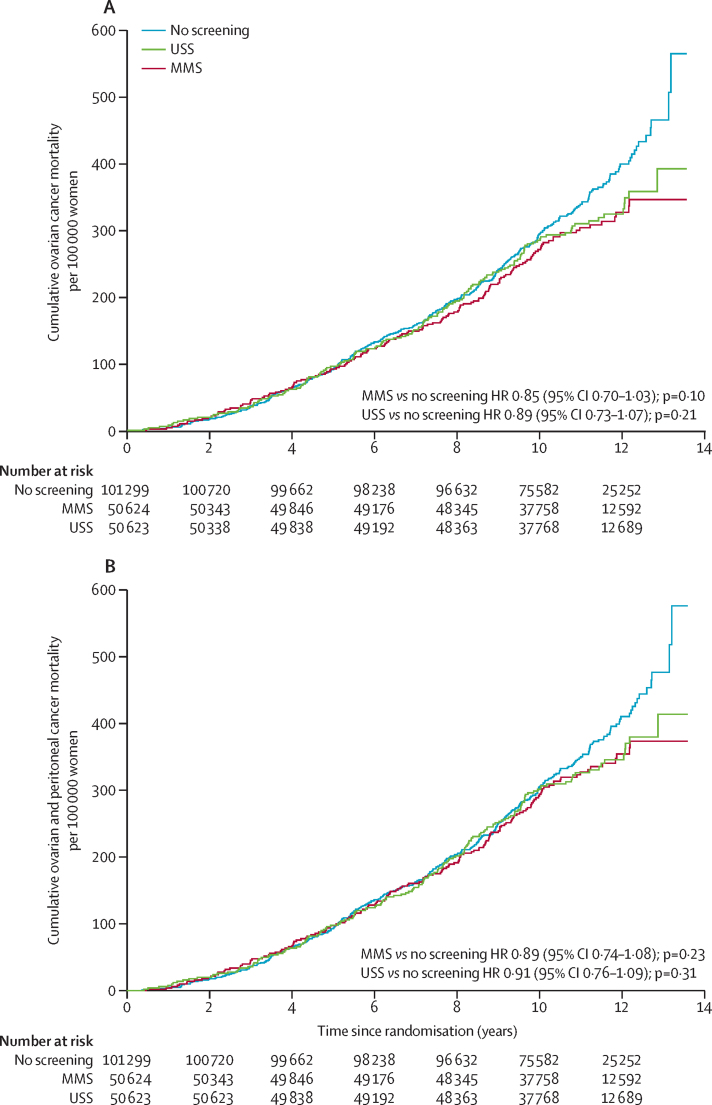
(A) Cumulative ovarian cancer and (B) ovarian and peritoneal cancer deaths The Royston-Parmar model is shown in the appendix (p 12, 13). HR=hazard ratio. MMS=multimodal screening. USS=ultrasound screening.

**Figure 3:**
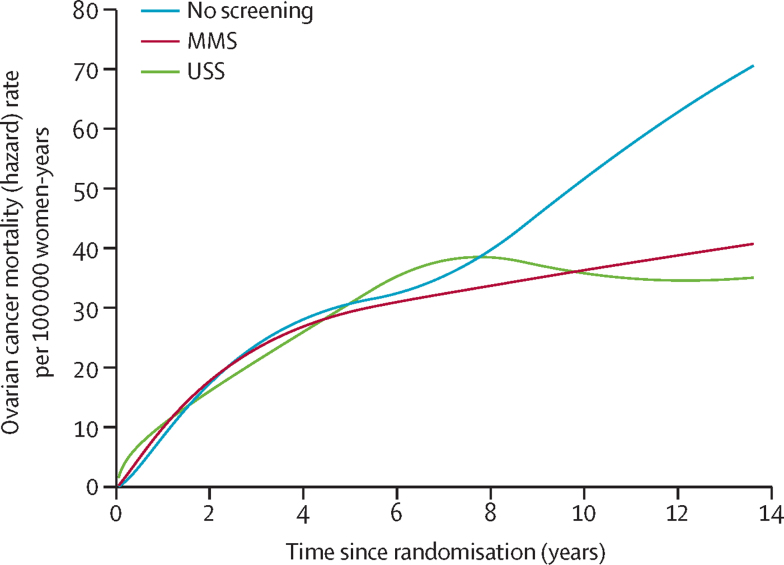
Rates of ovarian cancer The figure including confidence limits is in the appendix (p 14). MMS=multimodal screening. USS=ultrasound screening.

At censorship, 649 (0·32%) women had died of ovarian cancer: 347 (0·34%) in the no screening group, 148 (0·29%) in the MMS group, and 154 (0·30%) in the USS group. The mortality reduction over years 0–14 with the Cox model was 15% (95% CI −3 to 30; p=0·10) in the MMS group and 11% (−7 to 27; p=0·21) in the USS group (table 3). Figure 2 shows the Kaplan-Meier cumulative death rates; in the appendix (pp 12, 13), the ovarian curves are overlaid with RP fit. Figure 3 and the appendix (p 14) show that the no screening group hazard rate continues to rise throughout the study period, whereas the MMS hazard rate starts levelling off, becoming substantially lower than that of the no screening group at about 7 years, with the USS hazard rate levelling off at about 9 years, showing a potential delayed effect of screening. In the appendix (pp 15, 16), HRs plotted over time^32^ from the RP model, a non-parametric estimate of HRs, and averaged post-hoc HRs over 0–7 years and 7–14 years are shown. After year 7, the HRs decrease rapidly, showing non-proportional hazards (type II)^35^ and a delayed mortality reduction for years 7–14 of 23% (95% CI 1–46) for MMS and 21% (−2 to 42) for USS. For years 0–7, the estimated mortality reduction was much smaller: 8% (−20 to 31) for MMS and 2% (−27 to 26) for USS (figure 2, table 3).

**Table 3:** Summary of analyses of relative reduction of ovarian and primary peritoneal cancer deaths

		**Number of women (n)**	**Deaths (n)**	**Mortality reduction 0–14 years (%)**	**p value**	**Mortality reduction 0–7 years (%)**	**Mortality reduction 7–14 years (%)**
**Ovarian cancer (primary analysis)**							
Cox model							
	MMS	50 624	148	15% (−3 to 30)	0·10	..	..
	USS	50 623	154	11% (−7 to 27)	0·21	..	..
	No screening	101 299	347	..	..	..	..
Royston-Parmar model							
	MMS	50 624	148	16% (−1 to 33)	0·11	8% (−20 to 31)	23% (1 to 46)
	USS	50 623	154	12% (−6 to 29)	0·18	2% (−27 to 26)	21% (−2 to 42)
	No screening	101 299	347	..	..	..	..
Royston-Parmar model (excluding prevalent cases)							
	MMS	50 561	120	20% (−2 to 40)	0·021	8% (−27 to 43)	28% (−3 to 49)
	No screening	101 183	281	..	..	..	..
Weighted log-rank (post-hoc)							
	MMS	50 624	148	22% (3 to 38)*	0·023	..	..
	USS	50 623	154	20% (0 to 35)*	0·049	..	..
	No screening	101 299	347	..	..	..	..
**Ovarian and primary peritoneal cancer (secondary analysis)**							
Cox model							
	MMS	50 624	160	11% (−8 to 26)	0·23	..	..
	USS	50 623	163	9% (−9 to 24)	0·31	..	..
	No screening	101 299	358	..	..	..	..
Royston-Parmar model							
	MMS	50 624	160	11% (−7 to 28)	0·15	4% (−25 to 27)	18% (−5 to 40)
	USS	50 623	163	10% (−8 to 27)	0·27	2% (−26 to 26)	17% (−8 to 38)
	No screening	101 299	358	..	..	..	..
Royston-Parmar model (excluding prevalent cases)							
	MMS	50 561	131	16% (−6 to 35)	0·047	5% (−30 to 37)	24% (−7 to 45)
	No screening	101 191	298	..	..	..	..
Weighted log-rank (post-hoc)							
	MMS	50 624	160	18% (−1 to 34)*	0·064	..	..
	USS	50 623	163	17% (−3 to 33)*	0·097	..	..
	No screening	101 299	358	..	..	..	..

Data in parentheses are 95% CIs. MMS=multimodal screening. USS=ultrasound screening.

* Mortality reduction from hazard ratio weighted by pooled cumulative ovarian cancer mortality.

**Figure 4:**
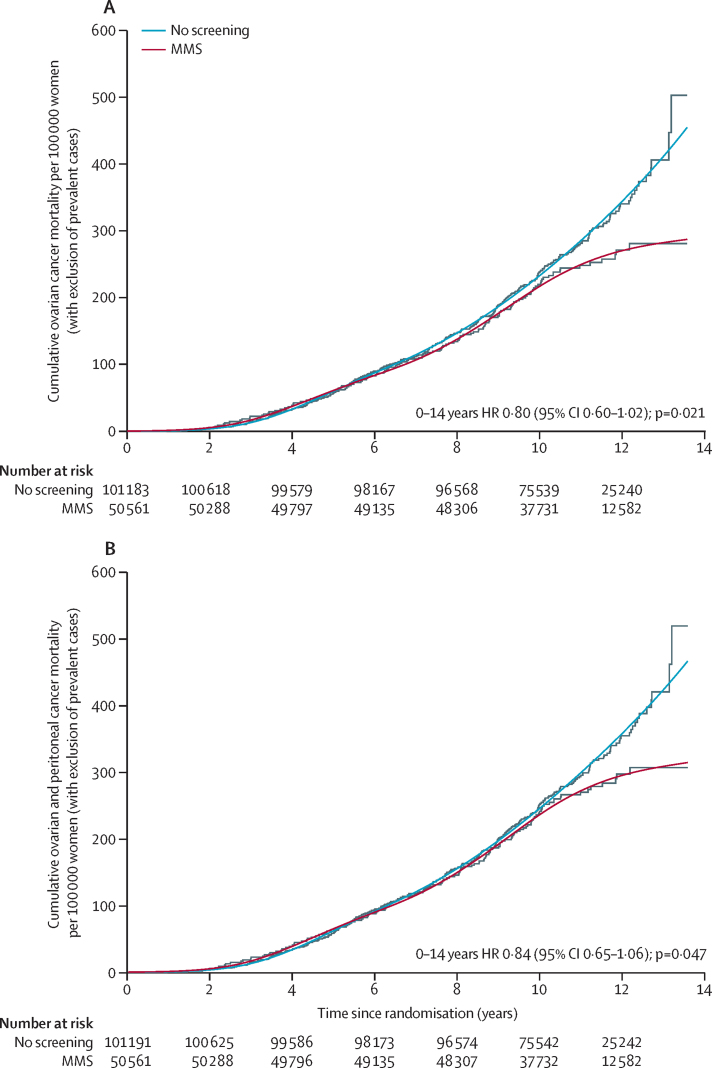
(A) Cumulative ovarian cancer and (B) ovarian and peritoneal deaths in MMS and no screening groups after exclusion of prevalent cases HRs and mortality reductions for 0–7 years and 7–14 years calculated from the Royston-Parmar model. Cumulative mortality curves from the Royston-Parmar model are overlaid onto Kaplan-Meier curves. HR=hazard ratio. MMS=multimodal screening.

In the MMS group, we excluded an average of 63 (19%) prevalent ovarian cases from the 338 cases and we excluded an average of 116 (18%) prevalent cases from the 630 cases in the no screening group, showing the expected equal proportion of prevalent cases in the two groups. Hazards were not proportional (p=0·037), so we fitted separate RP models to the survival data for each group, showing excellent overlap with the non-parametric Kaplan-Meier cumulative mortality curves (figure 4). The hazards between the MMS and no screening groups were significantly different (p=0·021), showing that ovarian cancer mortality was significantly lower in the MMS group (20% [–2 to 40]) than in the no screening group (table 3). The mortality reduction was also higher for years 7–14 (28% [–3 to 49]) than for years 0–7 (8% [–27 to 43]).

Because the HRs were not constant over time and the Cox model has low power for detection of a late effect of this type,^35^ we did a single post-hoc analysis using the WLR test inspired by the rationale in the PLCO Cancer Screening Trial report (appendix p 11).^24^ The median delay from randomisation to death in the no screening group was over 8 years (randomisation to cancer diagnosis 6 years; diagnosis to death 2·3 years). This analysis suggested a significant reduction in ovarian cancer mortality in the MMS group compared with the no screening group, but not in the USS group (table 3).

In addition to the ovarian and fallopian tube deaths, we noted a further 11 deaths due to primary peritoneal cancer in the no screening group, 12 in the MMS group, and nine in the USS group (table 3). This resulted in smaller mortality reductions with the MMS versus no screening and USS versus no screening comparisons than with the ovarian cancer mortality reductions (figure 2, table 3). As with the primary outcome, the no screening group hazard rate continues to rise, whereas the MMS and USS hazard rates start levelling off (appendix p 17). The RP model also yielded higher mortality reductions for years 7–14 than for years 0–7, with the MMS reduction increasing if prevalent cases are excluded (figure 4).

In the MMS group, of 427 448 eligible screening episodes, 345 570 were attended, giving a compliance of 80·8%. In the USS group, of 420 047 eligible screening episodes, 327 775 were attended, giving a 78·0% compliance (appendix p 6). At censorship with a maximum follow-up of 13·6 years, the preliminary number needed to screen to prevent one death from ovarian cancer was 641 (95% CI 375–1934; appendix p 18). Sensitivity analyses for source of ovarian cancer death, correlation within RCs, competing risks, and parametric estimation show only minor differences (appendix p 7).

We noted no evidence of a difference in deaths because of other causes between the MMS, USS, and no screening groups (appendix p 8). The age-standardised incidence and mortality rates for cancer and death from any cause increased with time from randomisation in the no screening group such that at the end of the trial, rates were similar to UK population rates (appendix p 23). Survival curves from date of diagnosis of ovarian cancers in the no screening group were similar to those of the age-standardised UK population, with a 5 year survival rate of 41% (95% CI 37–45) and a 10 year survival rate of 33% (28–38; appendix p 19).

The overall incidence of ovarian cancer was 57 (95% CI 53–62; 630 cancers; 1 097 089 women-years) per 100 000 women-years in the no screening group, 62 (55–68; 338 cancers; 548 553 women-years) per 100 000 women-years in the MMS group, and 57 (51–64; 314 cancers; 548 825 women-years) per 100 000 women-years in the USS group. We noted no evidence of a difference in incidence of ovarian cancer between the three groups (appendix pp 20–22). The incidence of invasive epithelial ovarian cancer and peritoneal cancer was also similar: 52 (48–61; 575 cancers; 1 097 089 women-years) per 100 000 women-years in the no screening group, 55 (48–57; 299 cancers; 548 553 women-years) per 100 000 women-years in the MMS group, and 47 (41–53; 259 cancers; 548 825 women-years) per 100 000 women-years in the USS group.

We noted screening complications in 30 (<1%) women in the MMS group and 61 (<1%) in the USS group, giving a screening-related complication rate of 8·6 per 100 000 in the MMS group and 18·6 per 100 000 in the USS group (appendix p 9). We noted benign adnexal pathology or normal adnexa in 488 (1·0%) women in the MMS group and 1634 (3·2%) women in the USS group who had screen-positive surgery (appendix p 9). This finding translates to 14 (345 572 annual screens) false-positive surgeries per 10 000 screens in the MMS group and 50 (327 775 annual screens) false-positive surgeries per 10 000 screens in the USS group. For each ovarian and peritoneal cancer detected by screening, an additional two (488 false positives; 212 ovarian and peritoneal cancers) women in the MMS group and ten (1634 false positives; 164 ovarian and peritoneal cancers) women in the USS group had false-positive surgery. Women in the MMS group had a complication rate of 3·1% (95% CI 1·7–5·0; 15 of 488) and those in the USS group had a rate of 3·5% (2·7–4·5; 57 of 1634). Outside of the trial, during the same period, 792 (0·8%) women in the no screening group, 466 (0·9%) in the MMS group, and 441 (0·9%) in the USS group had both ovaries or the only remaining ovary removed for a range of indications and had benign pathology or normal adnexa. Data for complications associated with these surgeries are not available. The overall ratio of women who had surgery resulting in benign adnexal pathology or normal adnexa to women with ovarian and peritoneal cancer was 1·2 (792:645) in the no screening group, 2·7 (954:354) in the MMS group, and 6·4 (2075:324) in the USS group. Details of final follow-up questionnaire responses are provided in the appendix (p 25). Ovarian cancer screening outside of UKCTOCS was documented in 1660 (4·3% [95% CI 4·1–4·5]) of 38 238 women in the no screening group who completed the follow-up questionnaire in 2014.

## Discussion

The relative mortality reduction was 15% in the MMS group and 11% in the USS group, but these reductions were not significant with the primary prespecified Cox analysis. In retrospect, it would have been preferable to specify a primary analysis that was weighted to reflect the predictable delay in mortality reduction in a screening trial of this type. Nevertheless, in view of the trend in mortality in the no screening and screening groups, significance using the Cox analysis could be achieved on further follow-up. However, the prespecified secondary subgroup analysis with exclusion of prevalent cases in the MMS group was significant, suggesting that the long-term effect of an MMS screening programme is about a 28% mortality reduction after 7 years of screening. This significant, yet delayed effect was supported by the sole post-hoc weighted analysis, which was also significant for the MMS group. These results are encouraging for various reasons. First, the mortality hazard rate in the no screening group seems to increase, whereas in the two screened groups, it levels off, resulting in decreasing HRs. This finding suggests that the difference in mortality between no screening and screening groups will increase with time and further follow-up. Second, the mortality difference was not constant across the trial. It was low for the first 7 years after randomisation, but the estimated mortality reductions increased during years 7–14. The mortality reduction in the MMS group for years 7–14 remained high in subgroup analyses with exclusion of prevalent cases for primary and secondary outcomes and in the secondary outcome analyses including peritoneal cancer deaths (albeit slightly lower). This late effect was predictable in view of the unavoidable time interval from randomisation to diagnosis and then death. For participants who died in the no screening group the median interval from randomisation to death was more than 8 years.

The late effect on mortality seen in this trial is often noted in screening trials in which survival is measured from date of randomisation. In the European Randomised Study of Screening for Prostate Cancer trial,^36^ an effect of screening only emerged 7 years after randomisation. Similarly, investigators of the Norwegian Colorectal Cancer Prevention Trial^37^ noted an effect 9 years from randomisation. The PLCO Cancer Screening Trial^24^, ^25^, ^26^, ^27^ and National Lung Screening Trial^28^ used the weighted log-rank test in anticipation of this delayed effect. Overall, the results suggested that an unequivocally significant difference in mortality might emerge after longer follow-up.

The estimate of relative reduction in ovarian cancer mortality in UKCTOCS is in keeping with relative reductions noted in breast cancer randomised screening trials, which vary between 15% and 25%, and with those noted in meta-analyses of observational studies, which vary between 13% and 17%.^38^ Using UK population data from 2007, Loberg and colleagues^38^ reported that for 1000 women invited to biennial mammography screening for 20 years from 50 years of age, two to three women are prevented from dying of breast cancer. Findings from this trial suggest that for 641 women screened annually using the multimodal strategy for 14 years, one ovarian cancer death is prevented.

The only directly comparable trial with this trial is the ovarian component of the PLCO Cancer Screening Trial.^24^ However, some differences exist between the two, including, in this trial, use of ROCA (as opposed to a CA125 cutoff) and detailed screening algorithms. We centrally managed implementation of the entire screening protocol with predefined pathways for all women detected to have abnormalities, fail-safe monitoring, and quality assurance. In the PLCO Cancer Screening Trial, no difference was noted in ovarian and peritoneal cancer deaths between the screening and control groups at a median follow-up of 12·4 years. In our secondary outcome analysis, we noted a mortality trend that was in keeping with our primary analysis and different to that noted in the PLCO Cancer Screening Trial. This finding follows on from the higher sensitivity and stage shift noted earlier in the MMS group of this trial.

Both venepuncture and transvaginal ultrasound were associated with minor complications and very low complication rates. Most postmenopausal women found transvaginal USS acceptable, with 3·5% reporting moderate or severe pain during the scan.^39^ As previously reported from the UKCTOCS psychosocial study,^15^ screening did not increase general anxiety. The number of women having surgery for false-positive screen results was as predicted in the trial design and for MMS, was much fewer than the upper acceptable limit, whereas for USS, it was on the border of acceptability. The small number of operations associated with MMS has been noted in previous trials.^9^, ^40^ During the course of this trial, the ratio of women who had surgery for which ovaries had benign pathology or were normal to those diagnosed with ovarian and peritoneal cancer was 2·3-times higher in the MMS group and 5·3-times higher in the USS group than in the no screening group. Complication rates of false-positive surgery of 3·1% in the MMS group and 3·5% in the USS group were similar to the major complication rate of 2·9% reported for benign surgery undertaken in NHS gynaecological oncology centres in a recent multicentre audit.^41^

Key strengths of this trial include scale, with more than 202 000 participants, more than 670 000 annual screening episodes, and more than 2·19 million women-years of follow-up; the multicentre setting within the UK NHS; central management of screening protocols with use of a bespoke web-based trial management system; high compliance; low contamination in the no screening group; completeness of ascertainment of deaths through linkage to national registries; an outcomes review committee masked to group assignment; and treatment of all women within the NHS. The healthy volunteer effect, noted initially, waned over time, such that by the end of the trial, age-standardised cancer incidence and all-cause mortality rates in the no screening group were similar to the UK population, as were ovarian cancer survival rates (appendix p 23).

The main limitation of this trial was our failure to anticipate the late effect of screening in our statistical design. Had we done so, the weighted log-rank test could have been planned in line with many other large cancer screening trials, including the ovarian component of the PLCO Cancer Screening Trial.^24^ As a result, we report, as planned, the Cox test as the primary analysis balanced by a significant preplanned analysis with exclusion of prevalent cases, with support from a sole post-hoc WLR test in the MMS group. Generalisability of the results will depend on central implementation of the screening protocols, with accompanying quality assurance processes. This implementation, although challenging, is achievable through the processes used in the NHS national screening programme. Further follow-up is needed to assess the extent of the mortality reduction before firm conclusions can be reached on the long-term efficacy and cost-effectiveness of ovarian cancer screening.

**This online publication has been corrected. The corrected version first appeared at thelancet.com on Jan 29, 2016**
