# Department of Error

**DOI:** 10.1016/S0140-6736(16)00228-2

**Published:** 2016-03-05

*Jacobs IJ, Menon U, Ryan A, et al. Ovarian cancer screening and mortality in the UK Collaborative Trial of Ovarian Cancer Screening (UKCTOCS): a randomised controlled trial.* Lancet *2015; **387:** 945–56*—In the ninth paragraph of the Results, the fourth sentence should have read “For each ovarian and peritoneal cancer detected by screening, an additional two (488 false positives; 212 ovarian and peritoneal cancers) women in the MMS group and ten (1634 false positives; 164 ovarian and peritoneal cancers) women in the USS group had false-positive surgery.” This correction has been made to the online version as of Jan 29, 2016, and has been made to the printed Article.

