# Supplementary material for: Ovarian cancer screening and mortality in the UK Collaborative Trial of Ovarian Cancer Screening (UKCTOCS): a randomised controlled trial
Source: Lancet. 2016 Mar 5;387(10022):945–56. doi: 10.1016/S0140-6736(15)01224-6 (PMC4779792; doi:10.1016/S0140-6736(15)01224-6)
Supplement: Supplementary appendix [file mmc1.pdf]

THE LANCET

Supplementary appendix

This appendix formed part of the original submission and has been peer reviewed. We post it as supplied by the authors.

Supplement to: Jacobs IJ, Menon U, Ryan A, et al. Ovarian cancer screening and mortality in the UK Collaborative Trial of Ovarian Cancer Screening (UKCTOCS): a randomised controlled trial. *Lancet* 2015; published online Dec 17. http://dx.doi.org/10.1016/S0140-6736(15)01224-6.

Appendix: Supplementary Material

Contents

A. Supplementary Tables 3
Web Table 1: ICD-10 Codes of Notes Reviewed by the Outcomes Committee 3
Web Table 2: Source of notification of cancer of women whose notes were submitted for Outcome Review 4
Web Table 3: Morphology and stage of ovarian and primary peritoneal cancers 5
Web Table 4: Compliance with annual screening 6
Web Table 5: Sensitivity analysis for primary analysis 7
Web Table 6: Original underlying cause of death as per Death Certificate excluding ovarian/primary peritoneal cancer by group 8
Web Table 7: Complications related to screening and screen-positive surgery in women with benign or normal adnexa 9
B. Supplementary Figures 10
Web Figure 1: Change point distribution in three cases with uniform AUC. 10
Web Figure 2: Overall ovarian cancer failure rate depicting the probability weighting scheme used for all weighted analyses. 11
Web Figure 3a: Cumulative ovarian cancer deaths by randomisation group with RP models overlaid - MMS versus no screening. 12
Web Figure 3b: Cumulative ovarian cancer deaths by randomisation group with RP models overlaid - USS versus no screening. 13
Web Figure 4: Ovarian cancer death rates with confidence limits by randomisation group. 14
Web Figure 5a: Non-parametric estimate of hazard ratio from smoothed scaled Schoenfeld residuals and parametric estimate from Royston-Parmar model with 95% confidence bands (showing close agreement) and estimates of average hazard ratio between 0-7 years and 7-14 years: MMS versus no screening. 15
Web Figure 5b: Non-parametric estimate of hazard ratio from smoothed scaled Schoenfeld residuals and parametric estimate from Royston-Parmar model with 95% confidence bands (showing close agreement) and estimates of average hazard ratio between 0-7 years and 7-14 years: USS versus no screening. 16
Web Figure 6: Rates of ovarian and peritoneal cancer by randomization group. 17
Web Figure 7: Mortality difference and numbers needed to screen (NNS) to prevent one death from ovarian cancer. 18
Web Figure 8: Ovarian cancer survival in women with ovarian cancer in the no screening group. Overlaid are UKCTOCS 5 and 10 year survival rates (red dash lines) and age-standardized UK population yearly survival rates (pink circles). 19

Web Figure 9: Cumulative incidence of ovarian cancer cases by randomization group. 20
Web Figure 10a: Cumulative incidence rates of ovarian cancer cases by randomization group. ... 21
Web Figure 10b: Cumulative incidence rates of ovarian cancer cases by randomization group with 95% confidence bands using Royston-Parmar models with 1 cubic spline knot for each group. 22
Web Figure 11: Standardized mortality ratio estimates (left panel) and standardized incidence ratio estimates (right panel) with 95% confidence limits over time for the no screening group. 23
C. Estimation of CA125 Change-point............ 24
D. Outcomes review 25
E. Details of final follow up questionnaire............ 25
F. UKCTOCS committees and teams 26

A. Supplementary Tables

ICD-10 code	Description
C56	Malignant neoplasm of ovary
C57·0	Malignant neoplasm of fallopian tube
C57·4	Uterine adnexa, unspecified
C57·7	Other specified female genital organs
C57·8	Malignant neoplasm of overlapping lesion of female genital organs
C57·9	Malignant neoplasm of female genital organ, unspecified
C48·0	Retroperitoneum
C48·1	Specified parts of peritoneum
C48·2	Malignant neoplasm of peritoneum, unspecified
C48·8	Overlapping lesions of retroperitneum and peritoneum
C76·2	Malignant neoplasm of abdomen
C76·3	Malignant neoplasm of pelvis
C80	Malignant neoplasm without specification of site
D07·3	Carcinoma in situ of other/unspecified female genital organ
D28·2	Benign neoplasm of fallopian tube
D28·9	Benign neoplasm of female genital organ, unspecified
D36·9	Benign neoplasm of unspecified site
D39·1	Neoplasm of uncertain or unknown behaviour of ovary
D39·9	Neoplasm of uncertain or unknown behaviour of female genital organ, unspecified

Abbreviations: ICD, International Classification of Diseases.

Web Table 1. ICD-10 Codes of Notes Reviewed by the Outcomes Committee

Source of notification	Women with source data available	Women with relevant ICD code/cancer*
Cancer Registry	45 877 (202 625 flagged)	**1474**
Death certificate	11 807 (202 625 flagged)	**1314**[†]
Hospital Episode Statistics‡	97 505 (158 077 before matching)	**572**
National Cancer Intelligence Network (NCIN)‡	32 846 (158 077 before matching)	**871**
Follow-up Questionnaire 1	13 0581	**171**
Follow-up Questionnaire 2	86 039	**211**
Contacted by patient or relative		**405**
Informed by physician / trial surgery		**442**
**Total number of women reviewed by Outcomes Committee**	**3110**	

Data is n. *C56, C57·0, C57·4, C57·7, C57·8, C57·9, C48·0, C48·1, C48·2, C48·8, C76·2, C76·3, C80, D07·3, D28·2, D28·9, D36·9, D39·1, and D39·9. †In absence of cancer registration. ‡Only available for women resident in England.

Web Table 2: Source of notification of cancer of women whose notes were submitted for Outcome Review

Characteristic	MMS	USS	No screening	Total
**Morphology**				
**Ovarian cancers**	**338**	**314**	**630**	**1282**
**Invasive epithelial ovarian/tubal/undesignated cancer**	**283**	**249**	**559**	**1091**
Type I invasive epithelial cancer	49	32	87	168
Low grade serous	12	7	17	36
Carcinoma, NOS	0	0	3	3
Mucinous	5	6	22	33
Endometrioid (low grade)	16	7	23	46
Clear cell	16	12	20	48
Brenner	0	0	1	1
Mixed	0	0	1	1
Type II invasive epithelial cancer	212	194	410	816
High grade serous	169	156	331	656
Carcinoma, NOS	19	20	34	73
High grade endometrioid	17	8	10	35
Carcinosarcoma	7	10	32	49
Adenocarcinoma	0	0	2	2
Neuroendocrine carcinoma	0	0	1	1
Type uncertain (grade unknown)	22	23	62	107
Serous	5	6	15	26
Carcinoma, NOS	16	17	46	79
Endometrioid	0	0	1	1
Small Cell Carcinoma	1	0	0	1
**Primary borderline epithelial neoplasm of ovary**	**44**	**53**	**62**	**159**
Serous	25	40	30	95
Mucinous	17	10	28	55
Endometrioid	2	1	1	4
Brenner	0	0	3	3
Mixed	0	2	0	2
**Primary non-epithelial neoplasm of ovary**	**11**	**12**	**8**	**31**
Granulosa cell	6	6	7	19
Carcinoid	1	3	0	4
Sarcoma	1	2	0	3
Sertoli-Leydig Cell	2	1	0	3
Squamous Cell Carcinoma	1	0	1	2
**Primary ovarian neoplasm (histology not available)**	**0**	**0**	**1**	**1**
**Primary peritoneal cancer**	**16**	**10**	**15**	**41**
Serous	13	8	12	33
Carcinoma, NOS	3	2	1	6
Carcinosarcoma	0	0	1	1
Clear cell	0	0	1	1
**Stage**				
**Ovarian cancers**	**338**	**314**	**630**	**1282**
**Invasive epithelial ovarian/tubal/ undesignated cancer**	**283**	**249**	**559**	**1091**
I	76	39	91	206
Ia	29	13	35	77
Ib	4	2	1	7
Ic	43	24	55	122
II	31*	19	45	94
IIa	8	6	13	27
IIb	10	5	16	31
IIc	12	8	16	36
III	142	141	314	597
IIIa	11	4	12	27
IIIb	30	21	38	89
IIIc	101	116	264	481
IV	33	50	108	191
Unable to stage	1	0	1	2
**Primary non-epithelial neoplasm of ovary**	**11**	**12**	**8**	**31**
I	10	10	7	27
II	0	0	1	1
III	1	1	0	2
IV	0	1	0	1
**Primary borderline epithelial neoplasm of ovary**	**44**	**53**	**62**	**159**
I	41	50	49	140
II	0	0	4	4
III	3	3	9	15
**Primary ovarian neoplasm (stage not available)**	**0**	**0**	**1**	**1**
**Primary peritoneal cancer**	**16**	**10**	**15**	**41**
IIb	1	0	1	2
III	14	9	10	33
IV	1	1	4	6
Data are number. *Unable to substage 1 case				

Web Table 3: Morphology and stage of ovarian and primary peritoneal cancers

Annual screen	Women due screen*		Women ineligible† for screen		Women eligible for screen		Women who attended screen		Compliance		Predicted compliance‡		Observed / predicted compliance	
	**MMS**	**USS**	**MMS**	**USS**	**MMS**	**USS**	**MMS**	**USS**	**MMS**	**USS**	**MMS**	**USS**	**MMS**	**USS**
1	50624	50623	10	67	50614	50556	49822	47955	98·4%	94·9%	100·0%	100·0%	0·98	0·95
2	50624	50623	265	1019	50359	49604	45893	44106	91·1%	88·9%	94·5%	91·0%	0·96	0·98
3	50624	50623	569	1458	50055	49165	43588	41951	87·1%	85·3%	87·5%	85·4%	1·00	1·00
4	50624	50623	898	1862	49726	48761	41669	40025	83·8%	82·1%	83·6%	81·9%	1·00	1·00
5	50624	50623	1275	2239	49349	48384	39925	38286	80·9%	79·1%	80·4%	78·8%	1·01	1·00
6	50624	50623	1660	2624	48964	47999	38283	36345	78·2%	75·7%	77·6%	76·0%	1·01	1·00
7	50622	50615	2064	3021	48558	47594	35170	32969	72·4%	69·3%	75·0%	72·7%	0·97	0·95
8	40430	40406	2043	2842	38387	37564	26091	23950	68·0%	63·8%	69·5%	66·5%	0·98	0·96
9	28235	28227	1666	2240	26569	25987	16878	15186	63·5%	58·4%	65·2%	61·2%	0·97	0·95
10	13864	13849	954	1278	12910	12571	7328	6334	56·8%	50·4%	61·0%	56·1%	0·93	0·90
11	2132	2115	175	253	1957	1862	923	668	47·2%	35·9%	54·5%	48·3%	0·87	0·74
**Total**	**439027**	**438950**	**11579**	**18903**	**427448**	**420047**	**345570**	**327775**	**80·8%**	**78·0%**	**82·0%**	**80·0%**	**0·99**	**0·98**

Data are numbers or percentages. *Based on anniversary of Randomisation date. †Dead/ovaries removed or ovarian cancer diagnosed prior to screen. ‡Four percent attrition year on year.

Web Table 4: Compliance with annual screening

Description	Group	No. of Women	Deaths	Unweighted		Weighted*	
				Mortality reduction (95% CI)	p-value	Mortality reduction (95% CI)	p-value
**Outcome death due to ovarian cancer**							
1) Events restricted to those with either death or cancer registration	MMS	50624	148	15·7 (-2·3, 30·6)	0·081	23·8 (4·4, 39·4)	0·020
	USS	50623	154	11·1 (-7·5, 26·5)	0·222	11·1 (0·0, 35·8)	0·057
	No screening	101299	347	0		0	
2) Accounting for potential within-centre correlation: by allowing different baseline hazard for each RC	MMS	50624	148	14·7 (-3·4, 29·6)	0·102	23·2 (3·7, 38·7)	0·022
	USS	50623	154	11·3 (-7·2, 26·7)	0·210	20·3 (0·4, 36·2)	0·046
	No screening	101299	347	0		0	
3) Accounting for potential within-centre correlation by use of cluster-robust standard errors	MMS	50624	148	14·7 (-3·9, 30·0)	0·115	23·2 (2·7, 39·4)	0·029
	USS	50623	154	11·3 (-13·7, 30·8)	0·344	20·3 (7·3, 40·8)	0·136
	No screening	101299	347	0		0	
4) Competing risks regression model treating ‘other deaths’ as competing risk rather than censored	MMS	50624	148	14·7 (-3·4, 29·6)	0·105	23·2 (3·7, 38·7)	0·022
	USS	50623	154	11·3 (-7·3, 26·6)	0·217	20·2 (0·3, 36·1)	0·047
	No screening	101299	347	0		0	
5) Parametric estimation: proportional hazards Weibull model†	MMS	50624	148	14·7 (-3·4, 29·6)	0·102	23·2 (3·7, 38·7)	0·022
	USS	50623	154	11·3 (-7·2, 26·6)	0·212	20·3 (0·4, 36·2)	0·046
	No screening	101299	347	0		0	

Data is n and % (95% CI). *Mortality reduction and CI’s from hazard ratio weighted by pooled cumulative ovarian cancer mortality. †For the Weibull model shape parameter p = 1·62 (95% CI: 1·51, 1·75) and baseline (log-hazard) rate = -9·56 (95% CI: -9·87, -9·27) [unweighted] and p=3·02 (95% CI: 2·80, 3·23) with baseline (log-hazard) rate = -13·62 (95% CI: -13·17, -14·07) [weighted]

Web Table 5: Sensitivity analysis for primary analysis

Original underlying cause of death listed on death certificate excluding ovarian/primary peritoneal (ICD-10 code)*	Group			RR†	p value
	No screening	MMS	USS		
Malignant neoplasm of uncertain origin (C80)	243 (22·1)	126 (23)	111 (20·2)	0·98	0·78
Other cancers - not ovarian/primary peritoneal or C80 (C00-C99‡)	3144 (286·6)	1589 (289·7)	1554 (283·2)	1·00	0·98
Diseases of the circulatory system (I00-I99)	1471 (134·1)	728 (132·7)	703 (128·1)	0·97	0·45
Diseases of the digestive system (K00-K99)	286 (26·1)	134 (24·4)	151 (27·5)	1·00	0·96
Diseases of the nervous system (G00-G99)	249 (22·7)	127 (23·2)	110 (20)	0·95	0·58
Diseases of the respiratory system (J00-J99)	578 (52·7)	310 (56·5)	286 (52·1)	1·03	0·60
Mental and behavioural disorders (F00-F99)	160 (14·6)	81 (14·8)	65 (11·8)	0·91	0·42
Other	493 (44·9)	263 (47·9)	269 (49)	1·08	0·22
Missing	34 (3·1)	18 (3·3)	13 (2·4)	0·91	0·71
**Total deaths**	**6658**	**3376**	**3262**	**0·99**	**0·65**
**Total women years**	**1097089**	**548533**	**548825**		

Data are n (death per 100,000 women years) *Cause of death was categorised according to the codes in the International Classification of Diseases, 10th Revision (ICD-10). †RR mortality rate ratio for no screening group vs MMS & USS combined. ‡Excluding C56, C57·0, C57·7, C57·9, C48·1, C48·2 and C80.

Web Table 6: Original underlying cause of death as per Death Certificate excluding ovarian/primary peritoneal cancer by group

Complications related to screening			
MMS		*USS*	
Complication type	No. of women	Complication type	No. of women
Bruising	13	Pain	20
Pain	8	Cystitis/infection	11
Haematoma	3	Discomfort	5
Fainting	1	Bruising	2
Cystitis/infection	1	Fainting	1
Other	4	Other	22
**Total**	**30**	**Total**	**61**
**Rate**	**8·6/100 000**	**Rate**	**18·6/100 000**
**Complications related to screen-positive surgery**			
MMS		*USS*	
Complication type	No. of women	Complication type	No. of women
Anaesthetic	1	Injury to hollow viscus (4 GI, 3 bladder, 4 ureter)	11
Injury to hollow viscus (2 GI, 1 bladder)	3	Haemorrhage	11
Haemorrhage	2	Anaesthetic/Myocardial Infarction	3
Deep Vein Thrombosis	1	Hernia	6
Bowel obstruction	4	Deep Vein Thrombosis/Pulmonary Embolism	3
Wound breakdown - total dehiscence	1	Wound breakdown	6
Significant ileus	1	Bowel obstruction	4
Uterine perforation	1	Wound/supravaginal haematoma	4
Infection	1	Infection	6
		Pain - ward readmission/further operation	3
**Total**	**15**	**Total**	**57**
**Rate**	**3·1% (15/488)**		**3·5% (57/1634)**

GI – Gastro Intestinal. In women who had more than one complication, the most serious was reported.

Web Table 7: Complications related to screening and screen-positive surgery in women with benign or normal adnexa

Web Figure 1: Change point distribution in three cases with uniform AUC.

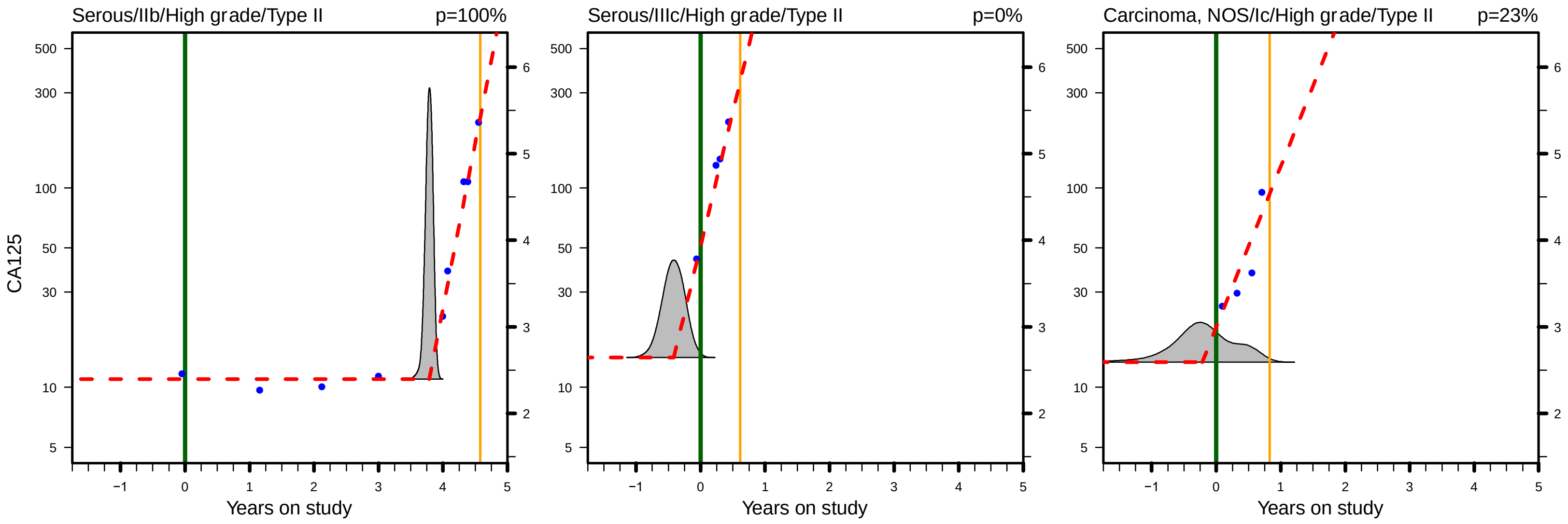

Change point distribution in three cases with green vertical line start of screening, gold vertical line is diagnosis, blue dots are CA125 test results (left vertical scale) and log(CA125) right vertical scale, red dashed line is best change-point model, and distribution of change-point is in grey where: (a) the change-point is after screening starts – case included in 100% of multiple imputation analyses, (b) the change-point is before screening starts and case is excluded from 100% of multiple imputation analyses, and (c) the change-point relative to start of screening is uncertain with 23% of multiple imputations including this case, since 23% of the change-point distribution is after the start of screening – right of green line.

Web Figure 2: Overall ovarian cancer failure rate depicting the probability weighting scheme used for all weighted analyses.

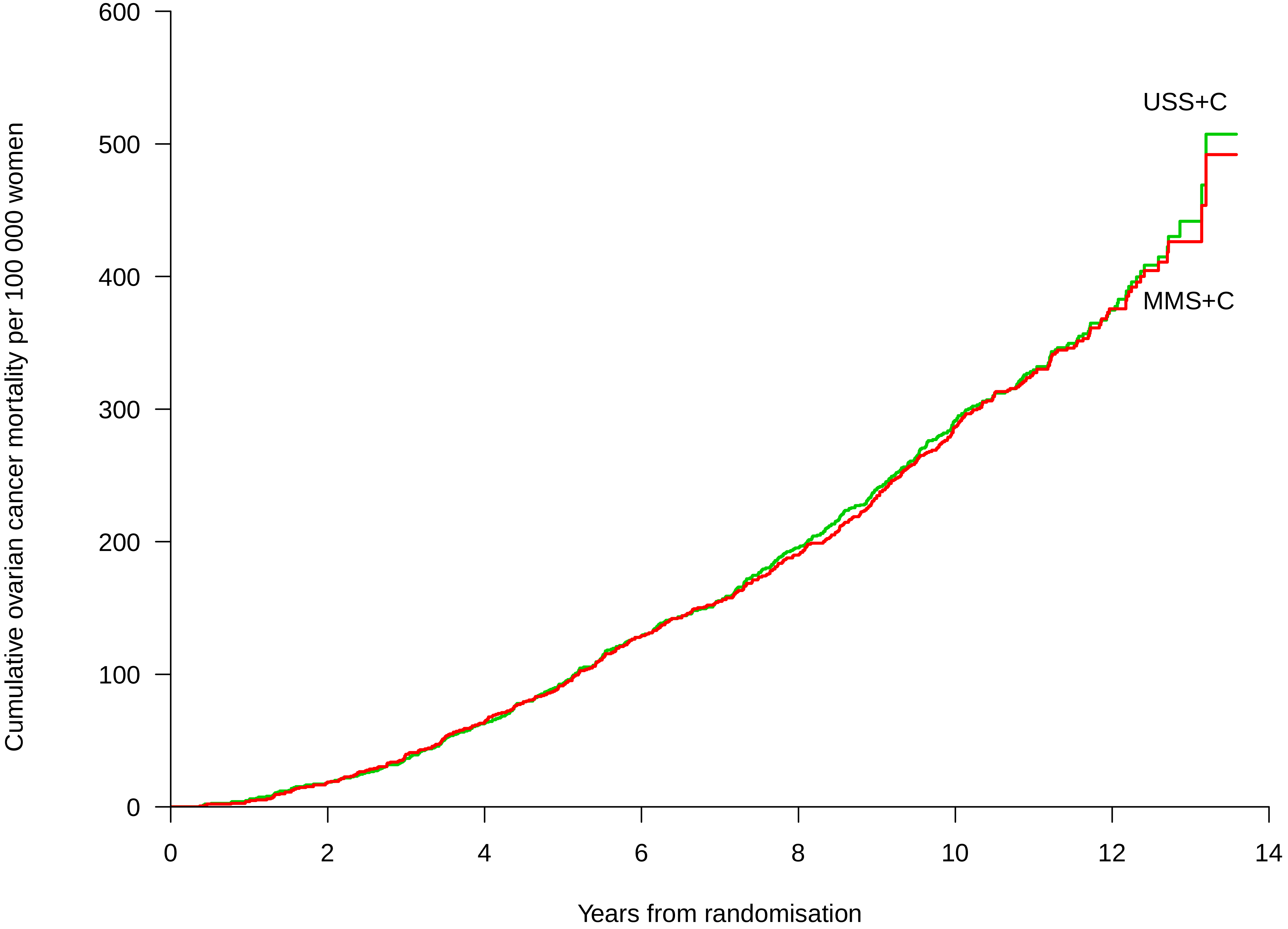

Web Figure 3a: Cumulative ovarian cancer deaths by randomisation group with RP models overlaid - MMS versus no screening (C).

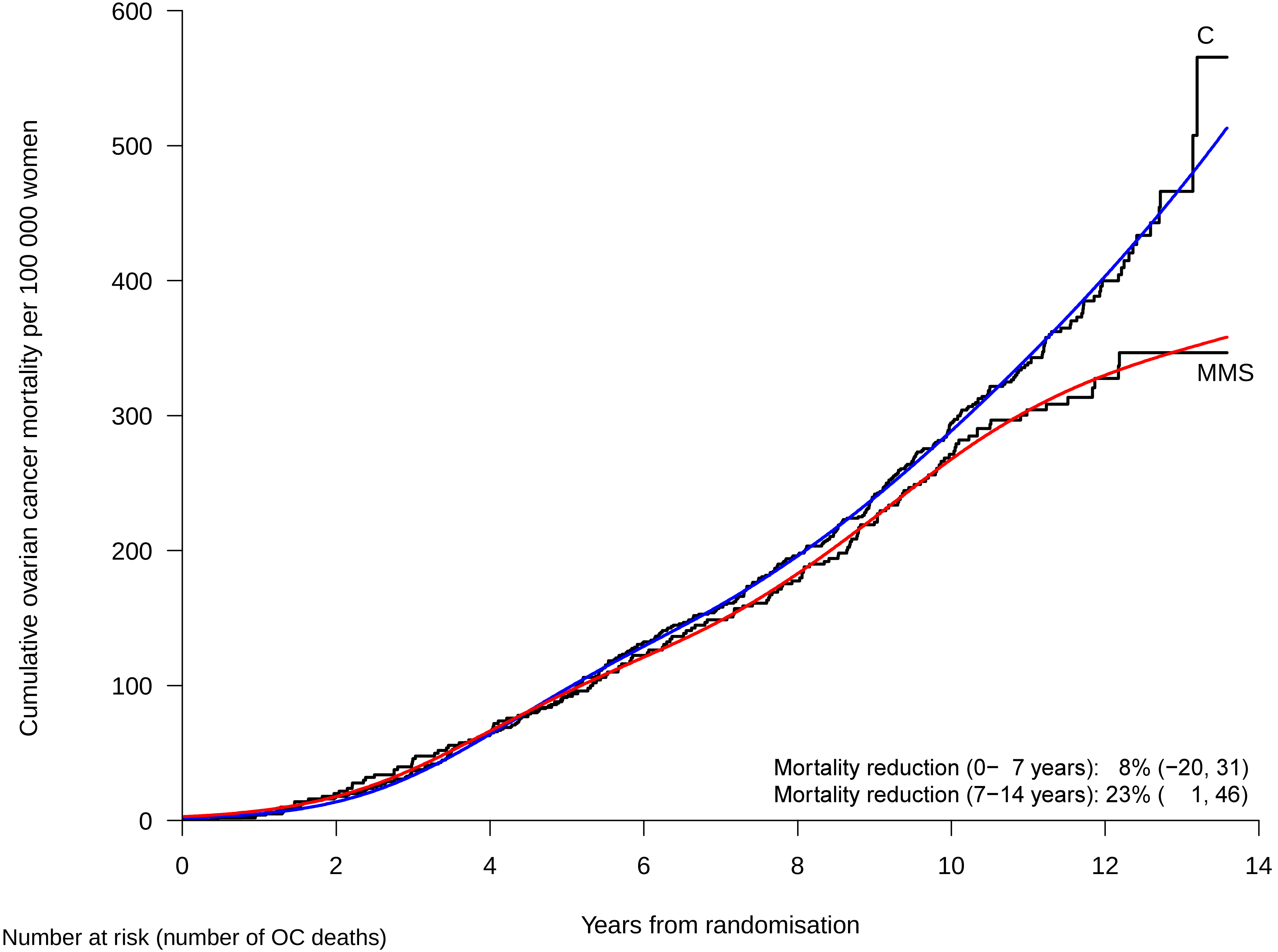

Number at risk (number of OC deaths)

C	101 299	(18)	100 720	(46)	99 662	(68)	98 238	(62)	96 632	(90)	75 582	(52)	25 252	(11)
MMS	50 624	(10)	50 343	(23)	49 846	(29)	49 176	(27)	48 345	(43)	37 758	(15)	12 592	(2)

Web Figure 3b: Cumulative ovarian cancer deaths by randomisation group with RP models overlaid - USS versus no screening (C).

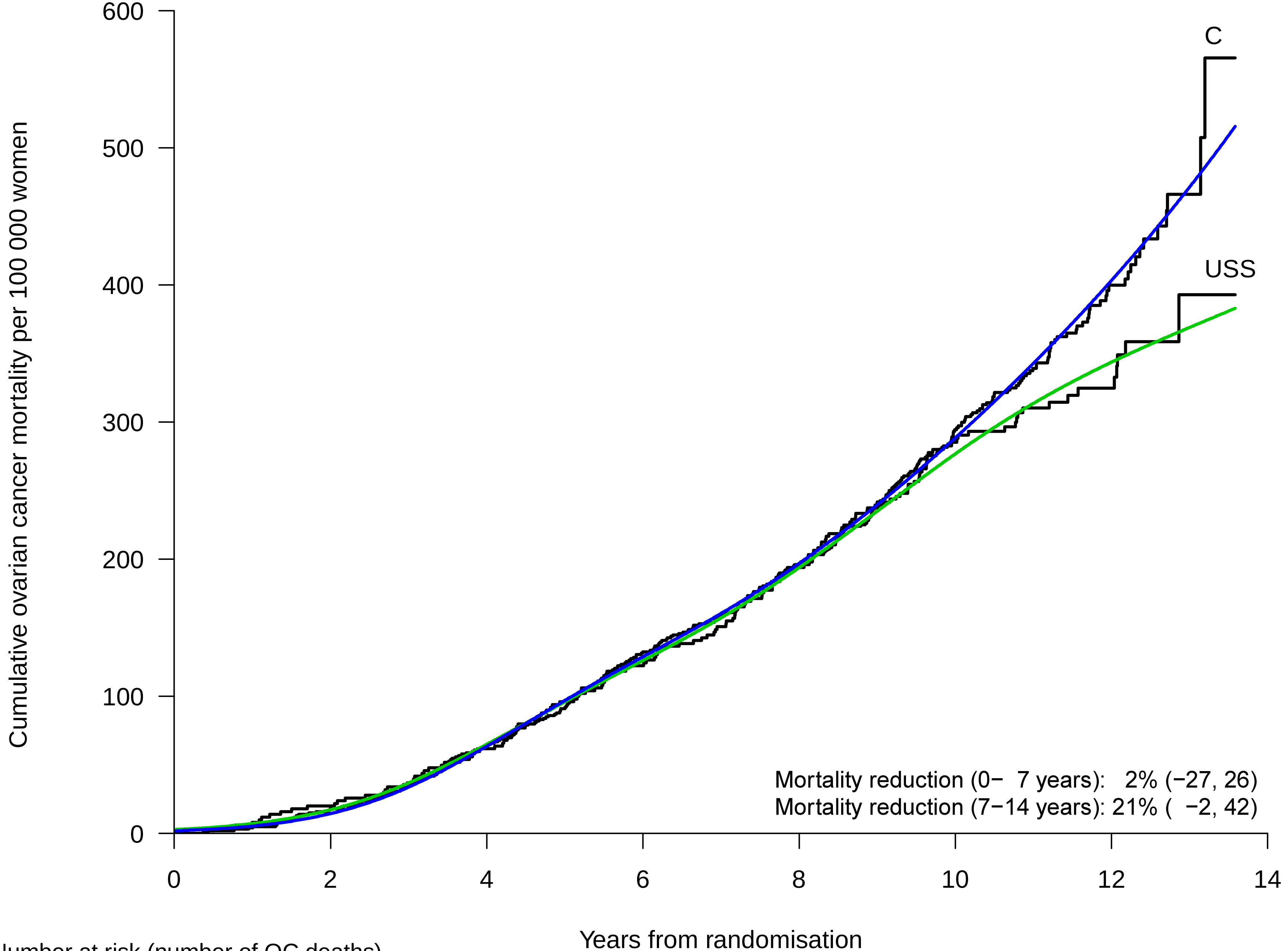

Number at risk (number of OC deaths)

C	101 299	(18)	100 720	(46)	99 662	(68)	98 238	(62)	96 632	(90)	75 582	(52)	25 252	(11)
USS	50 623	(10)	50 338	(21)	49 838	(30)	49 192	(35)	48 363	(42)	37 768	(11)	12 689	(5)

Web Figure 4: Ovarian cancer death rates with confidence limits by randomisation group. (C = No screening)

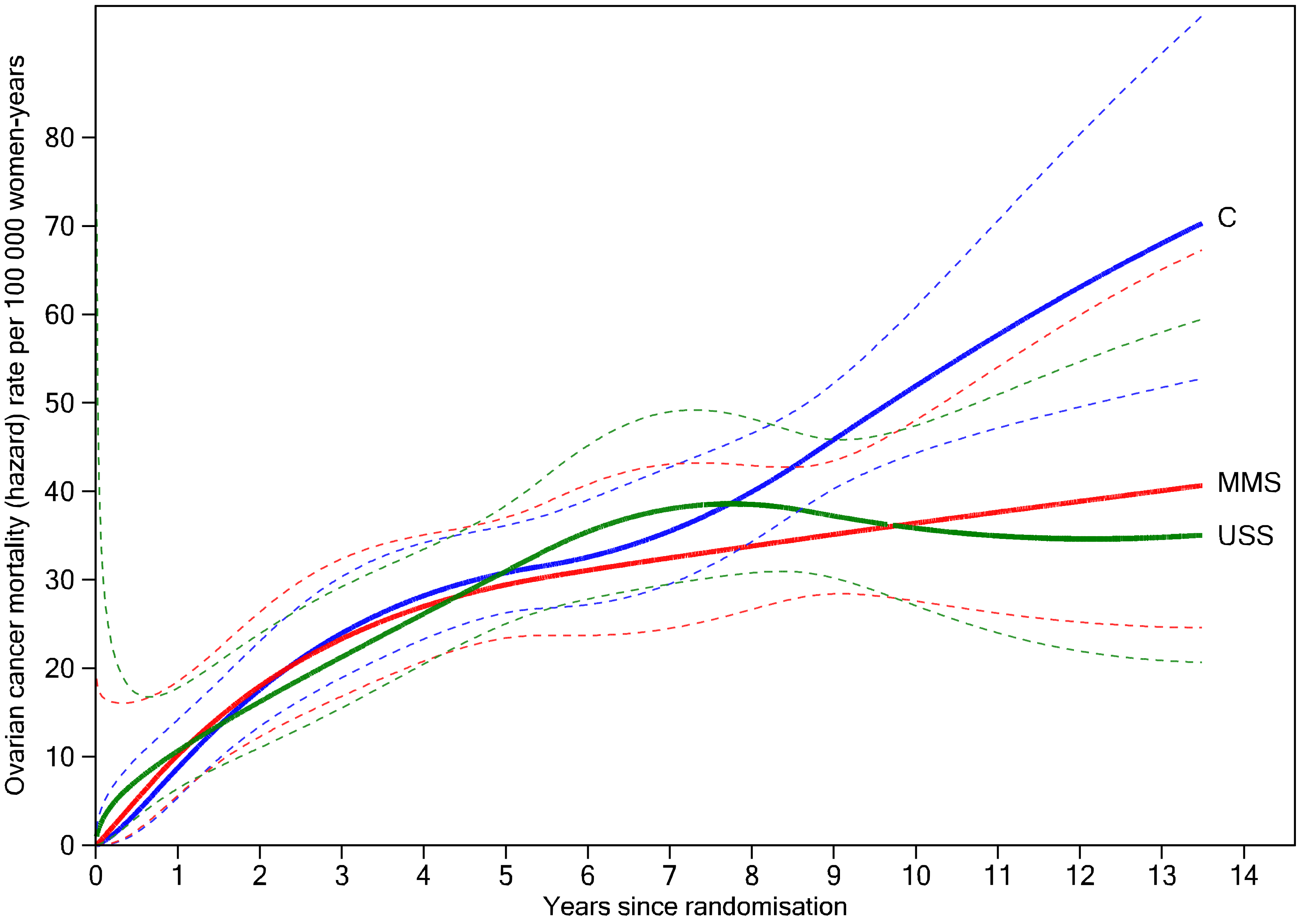

Web Figure 5a: Non-parametric estimate of hazard ratio from smoothed scaled Schoenfeld residuals and parametric estimate from Royston-Parmar model with 95% confidence bands (showing close agreement) and estimates of average hazard ratio between 0-7 years and 7-14 years: MMS versus no screening (C).

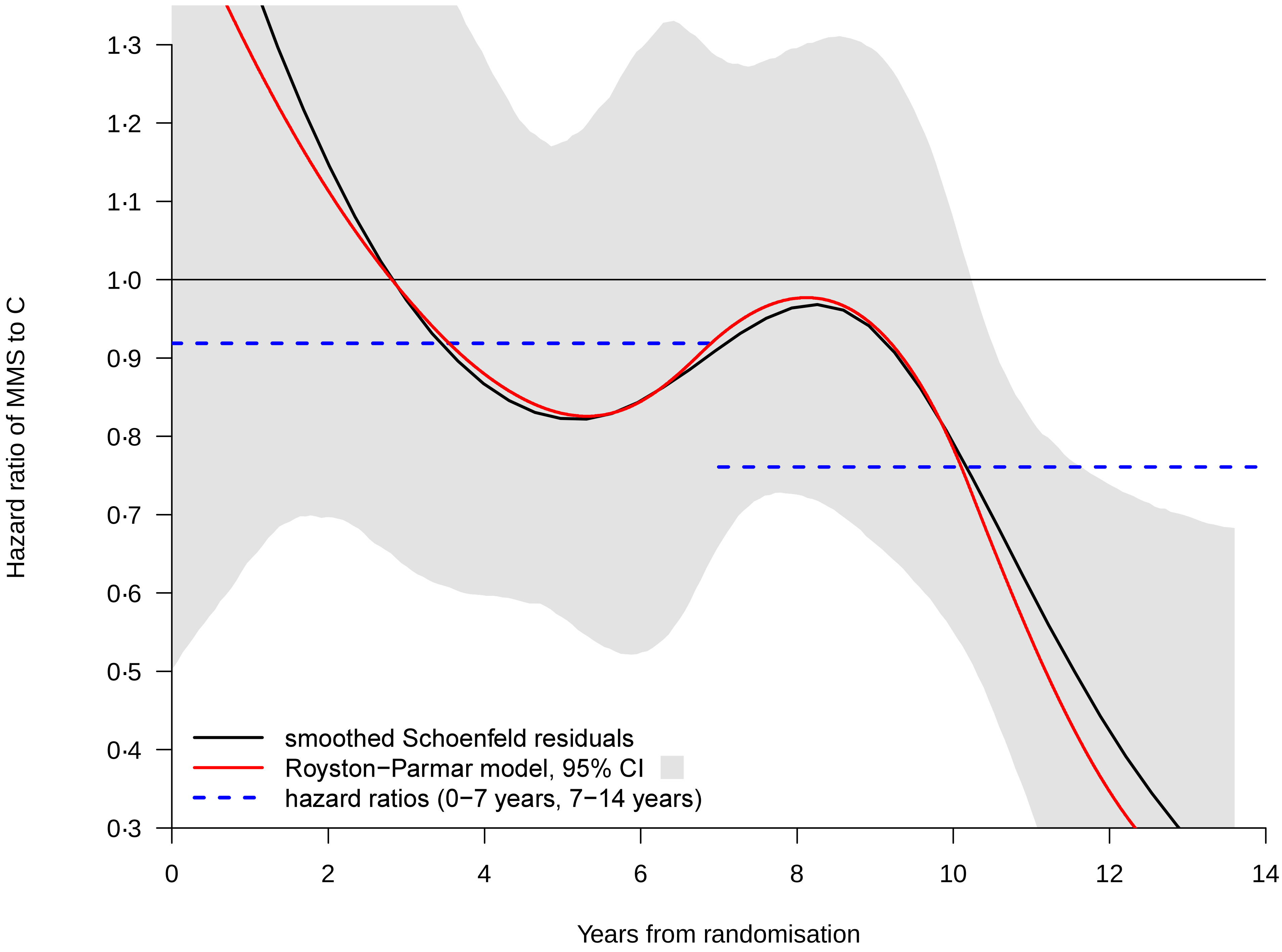

Web Figure 5b: Non-parametric estimate of hazard ratio from smoothed scaled Schoenfeld residuals and parametric estimate from Royston-Parmar model with 95% confidence bands (showing close agreement) and estimates of average hazard ratio between 0-7 years and 7-14 years: USS versus no screening (C).

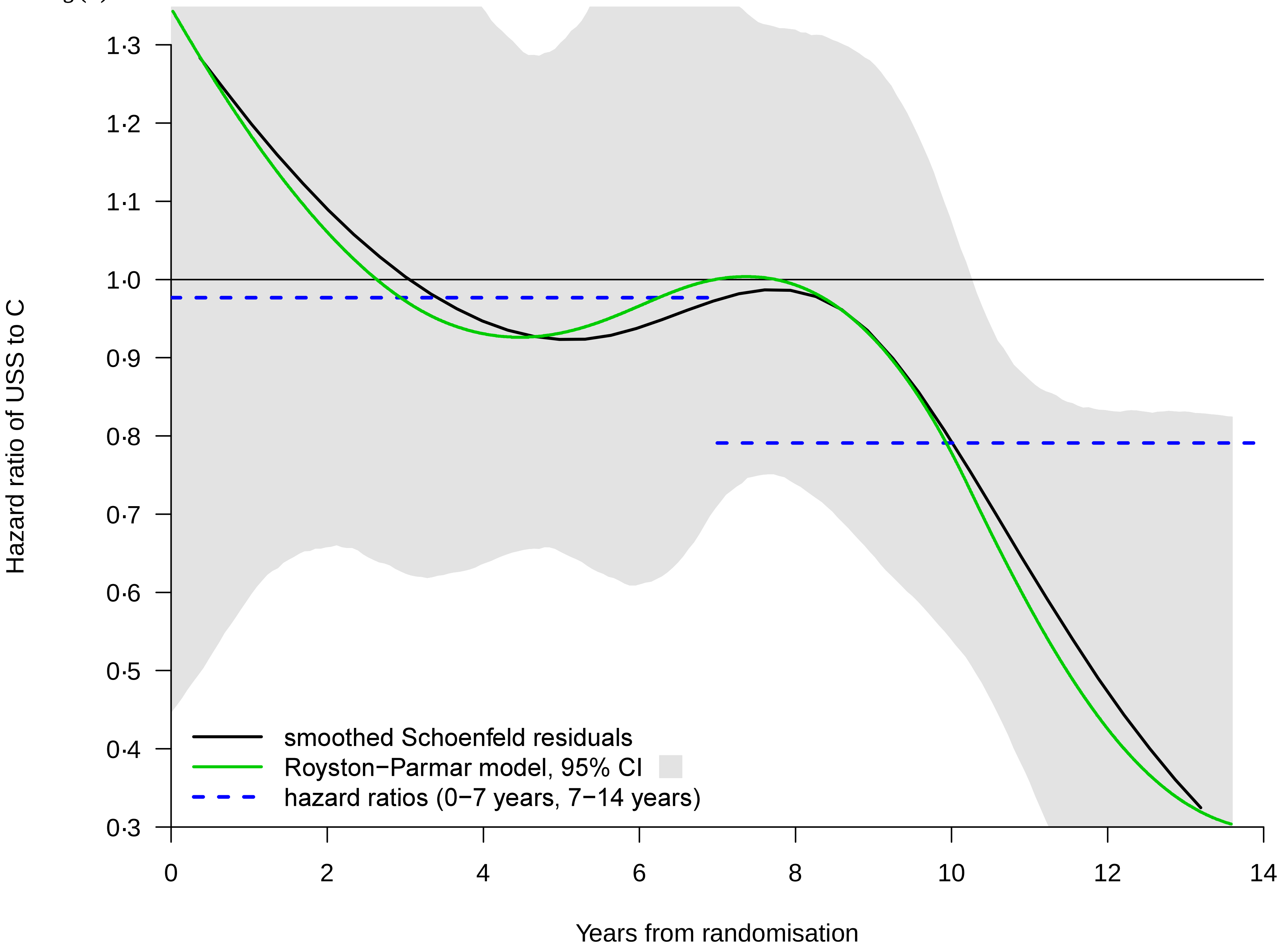

Web Figure 6: Rates of ovarian and peritoneal cancer by randomization group. (C = no screening)

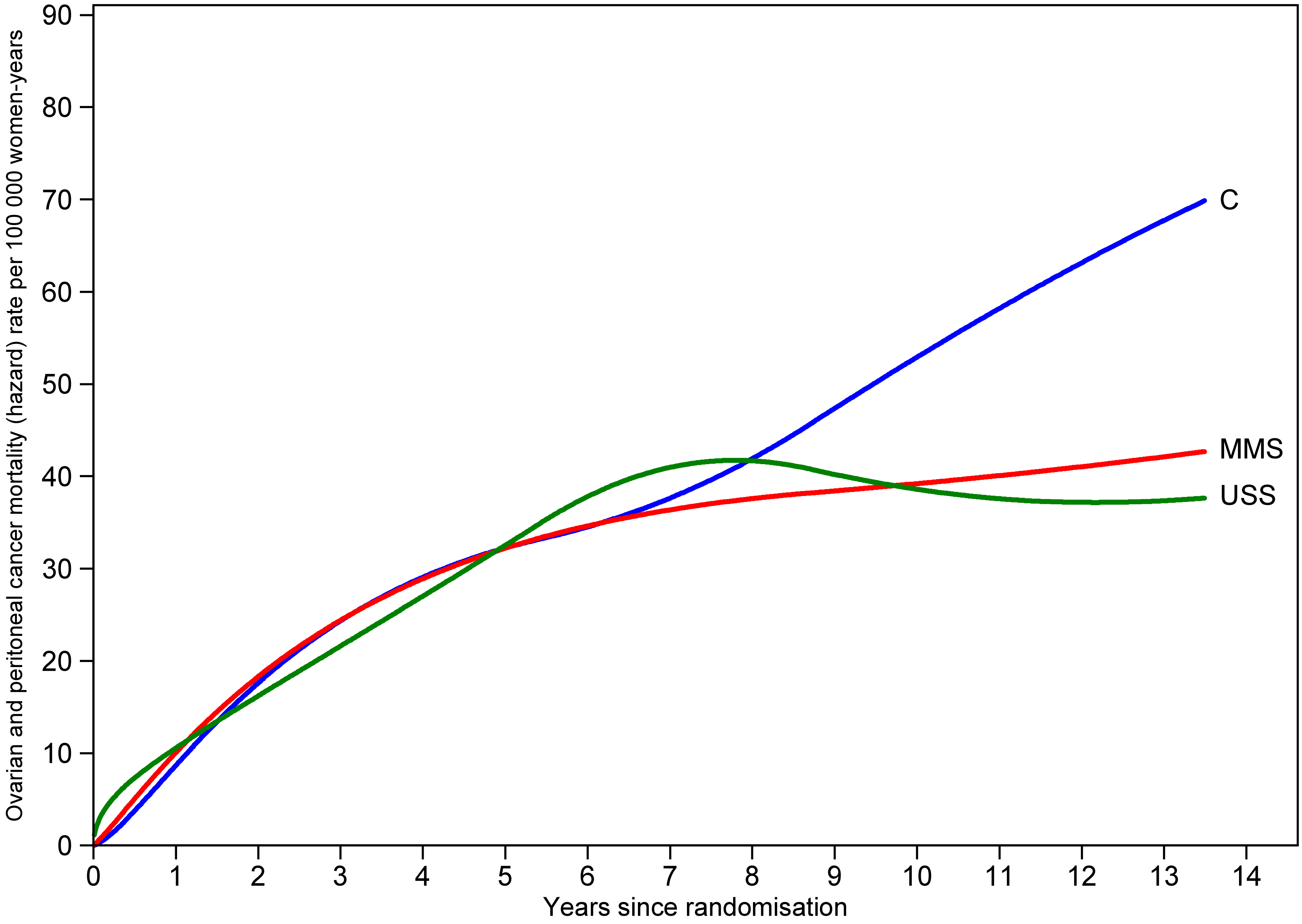

Web Figure 7: Mortality difference and numbers needed to screen (NNS) to prevent one death from ovarian cancer. (C = no screening)

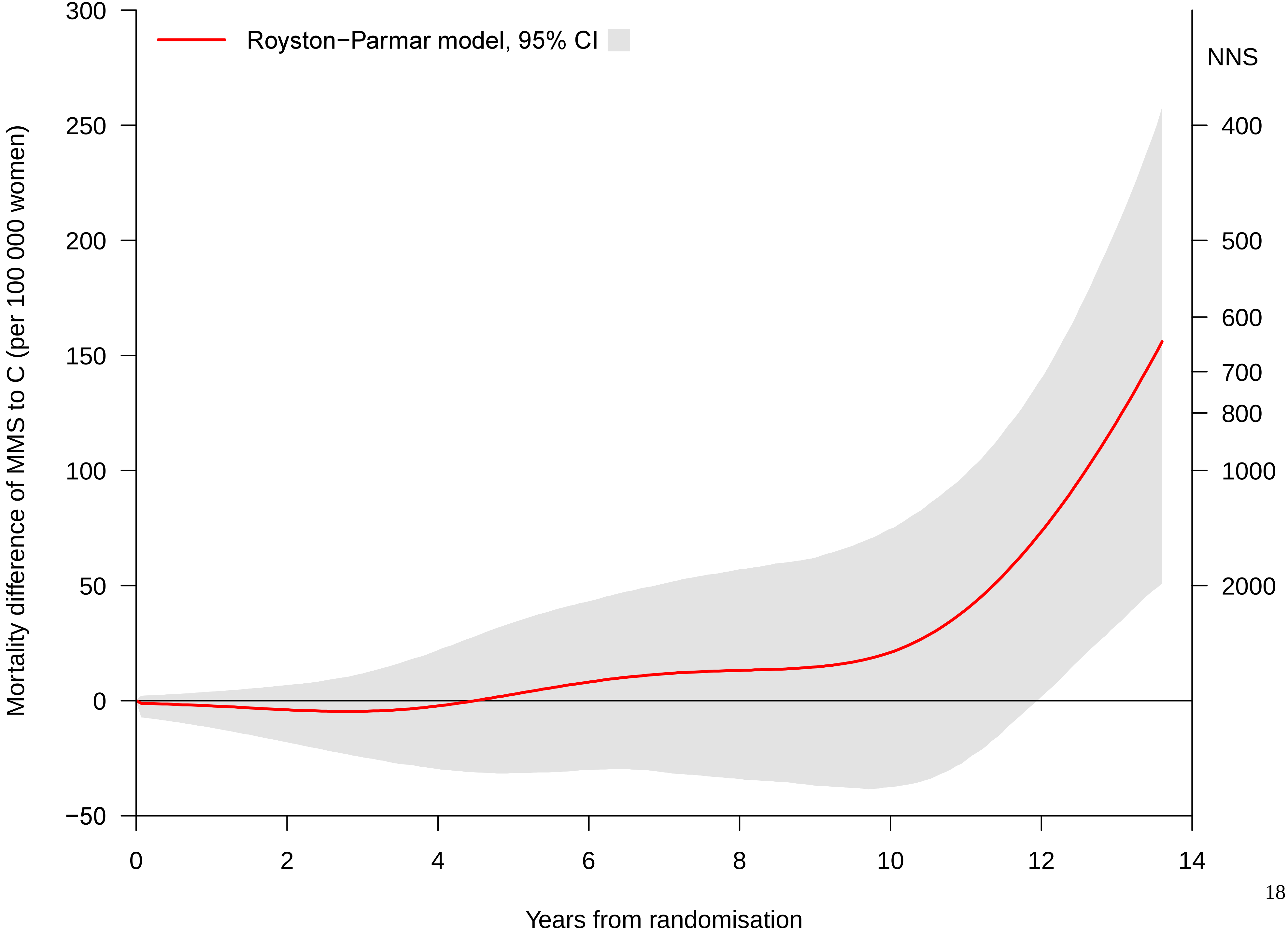

Web Figure 8: Ovarian cancer survival in women with ovarian cancer in the no screening group. Overlaid are UKCTOCS 5 and 10 year survival rates (red dash lines) and age-standardized UK population yearly survival rates (pink circles).

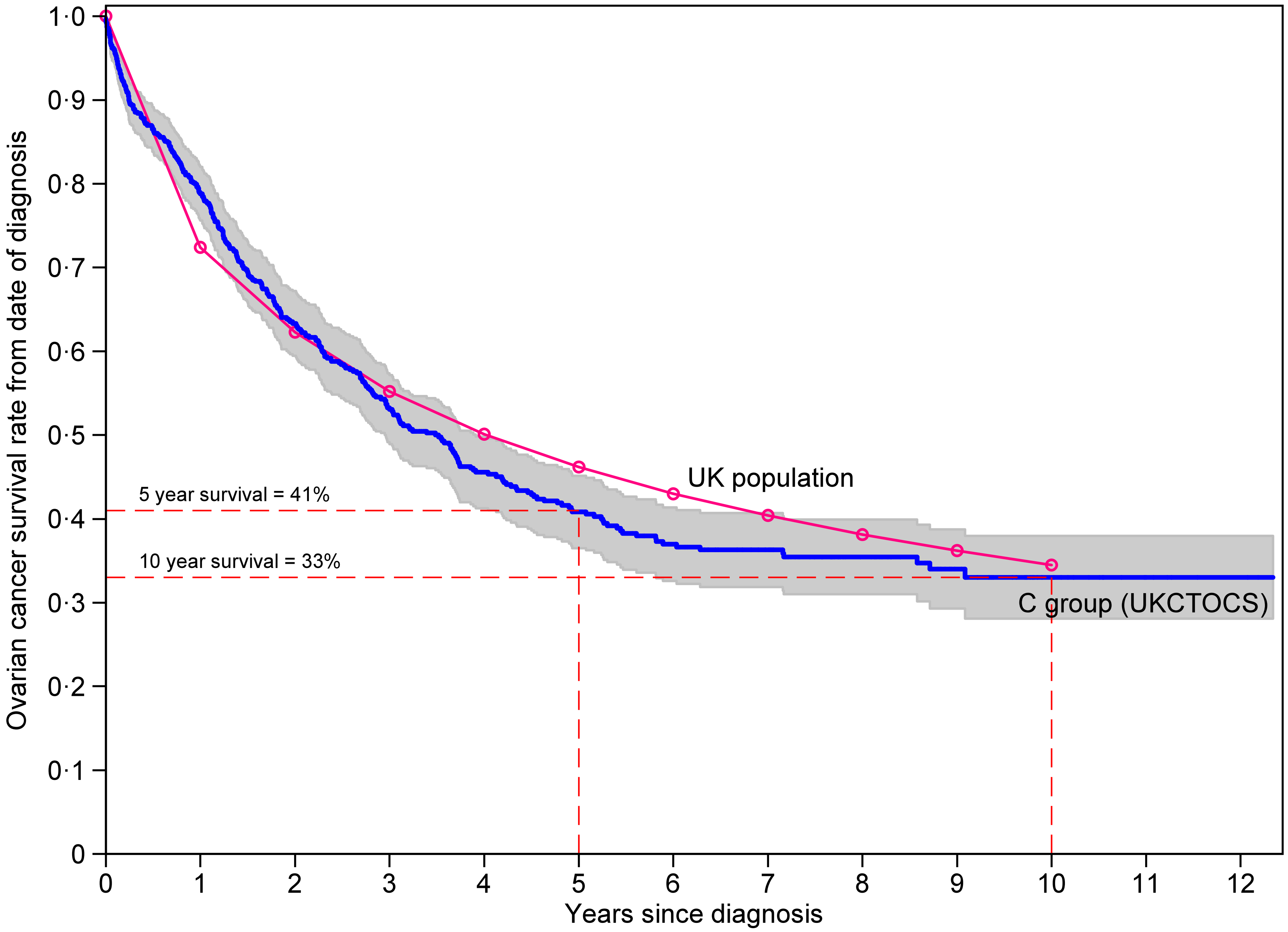

Web Figure 9: Cumulative incidence of ovarian cancer cases by randomization group. (C = no screening)

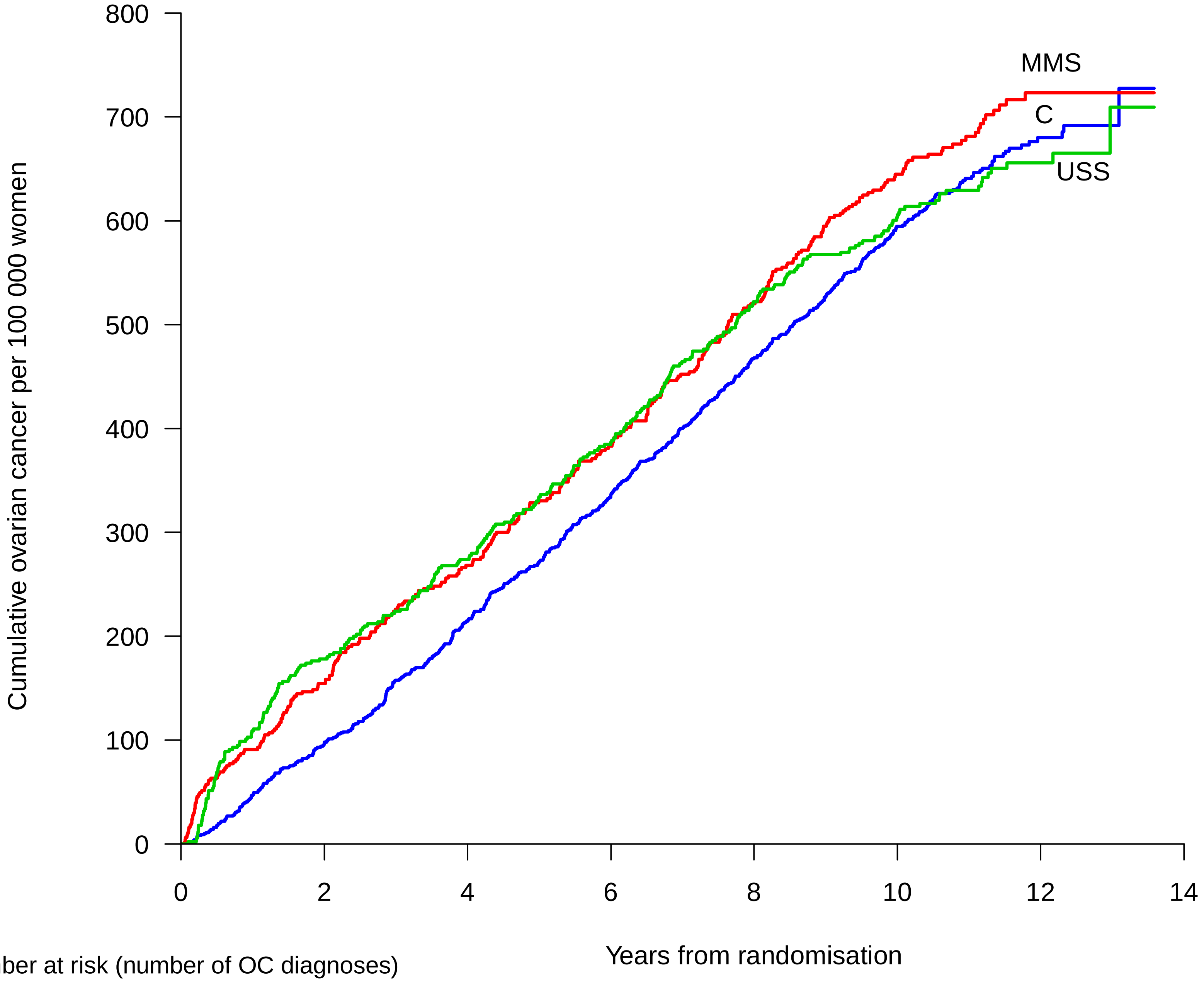

Number at risk (number of OC diagnoses)

C	101 299	(98)	100 640	(118)	99 508	(121)	98 032	(128)	96 358	(116)	75 346	(46)	25 168	(3)
MMS	50 624	(78)	50 271	(57)	49 735	(57)	49 040	(67)	48 175	(58)	37 616	(21)	12 535	(0)
USS	50 623	(90)	50 255	(48)	49 727	(56)	49 053	(66)	48 199	(37)	37 642	(15)	12 635	(2)

Web Figure 10a: Incidence rates of ovarian cancer cases by randomization group. (C = no screening)

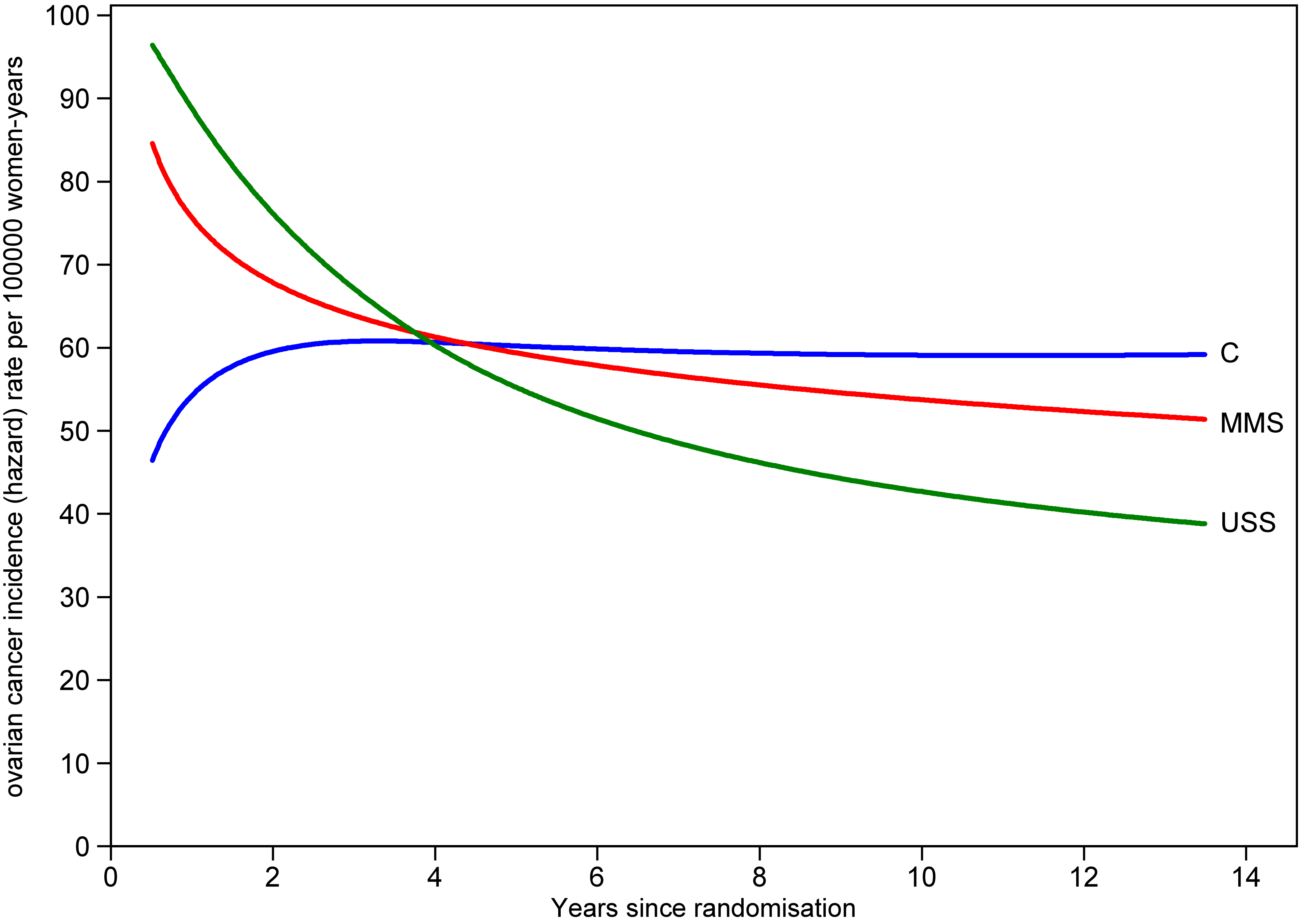

Web Figure 10b: Incidence rates of ovarian cancer cases by randomization group with 95% confidence bands using Royston-Parmar models with 1 cubic spline knot for each group. (C = no screening)

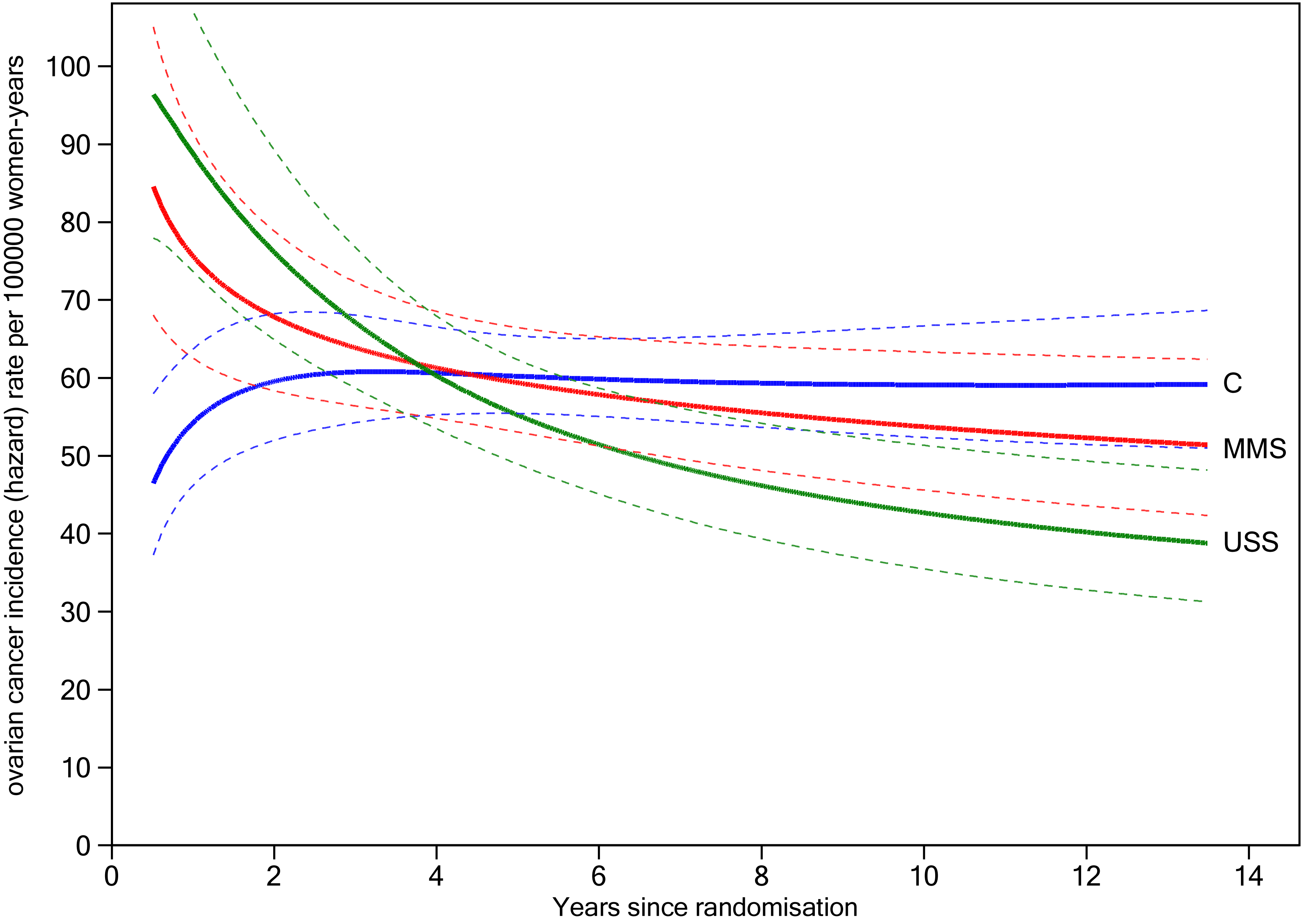

Web Figure 11: Standardized mortality ratio estimates (left panel) and standardized incidence ratio estimates (right panel) with 95% confidence limits over time for the no screening group. Observed rates have been compared to age-adjusted expected rates based on UK population data. Note, for presentational purposes, the upper-bound for the 13th year SMR estimate has been capped at 2·2, but is actually 2·88.

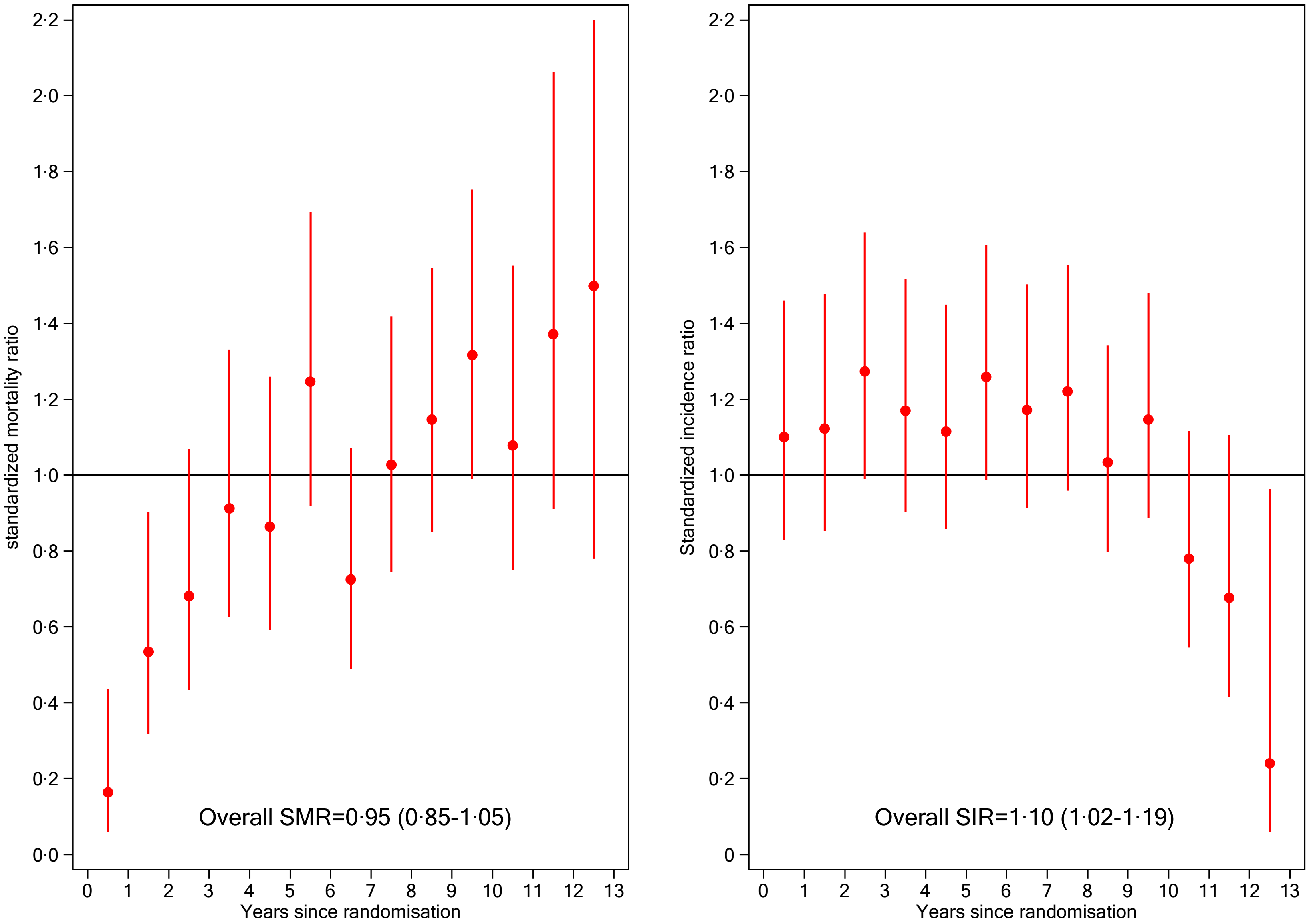

C. Estimation of CA125 Change-point

To identify the likelihood of a case being prevalent, we used the serial CA125 pattern to estimate whether each cancer's change-point occurred prior to randomisation (T_0). The intersection of the baseline CA125 line and the rising CA125 line estimated the change-point (Supplementary Figure 1a).[1] Cases where the change-point was before T_0 were excluded (Supplementary Figure 1b). This approach provides an objective assessment of which cases are prevalent based on a case's CA125 profile instead of assuming an arbitrary interval of time from T_0 to diagnosis to define prevalent cases. To estimate the change-point for ovarian cases in the no screening group, banked baseline serum samples were available in 517 of the 630 women. These were retrieved and CA125 measured in the CC laboratory using the same assay/analyser as during the trial. 105 baseline MMS samples were also re-assayed for CA125 revealing no significant difference due to long term storage between the new result and the measurement at randomisation. The results combined with CA125 at diagnosis in no screening group cases and the distribution of rate of rise from MMS cases were used to estimate change-points for no screening group cases using the same method as for the MMS cases.[1] For the no screening group cases and some MMS cases, there was uncertainty as to whether the change-point occurred after T_0 (Supplementary Figure 1c). Multiple imputation[2] of the change-points with 100 imputations accounted for this uncertainty. RP methods[3] modelled the cumulative hazards for each arm as a smooth spline function of time with the number of equally spaced knots chosen to minimize the Akaike information criterion (AIC).[3] With an RP model we assessed proportionality of hazards and effect of screening with Wald tests[3] combined over multiple imputations[2].

References

1. Skates SJ, Pauler DK, Jacobs IJ. Screening based on the risk of cancer calculation from Bayesian hierarchical changepoint and mixture models of longitudinal markers. *Journal of the American Statistical Association* 2001; **96**(454): 429-39.

2. Rubin DB. Multiple imputation for nonresponse in surveys. New York: John Wiley & Sons, Inc; 1987.

3. Royston P, Parmar MK. Flexible parametric proportional-hazards and proportional-odds models for censored survival data, with application to prognostic modelling and estimation of treatment effects. *Statistics in medicine* 2002; **21**(15): 2175-97.

D. Outcomes review

Ascertainment of outcomes involved interrogation of all available data sources to identify women diagnosed post-randomisation with any of 19 ICD-10 codes14 (Supplementary Table1). Copies of medical notes were retrieved for all except women who had an ICD10-C80 (malignant neoplasm of uncertain origin) and also had another specific non ovarian/peritoneal cancer registration. The table below details the proportion of women for whom we were able to provide information to the Outcomes review committee in the form of copies of medical notes in addition to cancer and/or death registration. Excluding women with ICD10-C80 (malignant neoplasm of uncertain origin) who also had another specific non ovarian/peritoneal cancer registration, we were able to provide additional information in 99% (1757/1767) of women with regards to cancer diagnosis and 97% (876/900) with regard to death diagnosis. There was no significant differences in the proportion of women with missing data between the randomisation groups.

Availability of information in addition to cancer and/or death registration

Review for cancer diagnosis

	Cases submitted to outcomes review			
	**No screening**	**MMS**	**USS**	**Total**
Total	812	495	460	1767
Additional data available	806	493	458	1757
No additional data	6 (0.74%)	2 (0.40%)	2 (0.43%)	10 (0.57%)
Comparison with no screening group (p value)		0.718	0.718	

Review for Death diagnosis

**Details**	Cases submitted to outcomes review			
	**No screening**	**MMS**	**USS**	**Total**
Total	450	234	216	900
Additional data available	439	229	208	876
No additional data	11 (2.44%)	5 (2.14%)	8 (3.70%)	24 (2.67%)
Comparison with no screening group (p value)		0.801	0.61	

E. Details of final follow up questionnaire

In 2014 the final follow-up questionnaire was sent to 169 762 (88 743 no screening group, 41 556 MMS, 39 463 USS) women. Questionnaires were not sent to 32 784 (12 556 no screening group, 9 068 MMS, 11 160 USS) women as they had died (12 035), been diagnosed with ovarian cancer (156) or requested not to be contacted (20 593). The response rate was 43·1% (38 238/ 88 743) in the no screening group; 55·3% (22 975/41 556) in the MMS group and 56·5% (22 297/39 463) in the USS group.

F. UKCTOCS committees and teams

DMEC Committee

Prof P Boyle (Chair), Prof APM Heintz, S Kjaer, EL Trimble.

Trial Steering Committee

Prof D Luesley (Chair; independent member), L Bayne (independent member) , Prof J Cuzick (independent member), Prof L Fallowfield, Prof I Jacobs, Prof U Menon, Prof M Parmar, Prof J Patnick (independent member).

Trial Management Committee

Prof I Jacobs (Chair), M Burnell, S Campbell, S Davies, Anne Dawnay, A Gentry-Maharaj, J Kalsi, Prof Lesley Fallowfield, Prof A McGuire, Prof U Menon, T Mould, Prof M Parmar, A Ryan, S Skates, R Woolas.

Ultrasound Sub-Committee

Prof U Menon (Chair), Prof N Amso, C Brunell, Prof S Campbell, G Fletcher, K Ford, A Gentry-Maharaj, J Kalsi, R Rangar, A Ryan, M Seif, G Turner.

Outcome Review Committee

N Singh (chair), E Benjamin, K Reynolds, , Prof M Widschwendter.

Coordinating Centre Team

U Menon (Lead), M Ahmad , T Akbar, N Alves, S Apostolidou, M Bacon, C Brunell, M Burnell, J Chapman, D Crump, J Cunningham, L Danquah, S Davies, A Dawnay, A Dyer, J Ford, A Gentry-Maharaj, A Gibson, T Goodall, S Grant, R Gunu, M Habib, L Hadcocks, R Hallett, N Hinkey, J Kalsi, C Karpinskyj, J Kerkhoff, Z Khan, S Lewis, W Liston, S Mohamed, L Odunlami, M Pamboris, S Philpott, T Roberts, A Ryan, A Sharma, J Sheals, K Sibley, C Spicer, S Spicer, L Sterry, C Stubbs, K Tamm, J Taylor, F Warburton, Y Wold, T Roberts.

Regional Trial Centre Teams

Gateshead

K Godfrey (Lead), A Lopes (Lead), J Callaghan, G Dorman, J Gibson, C Green, A Guest, A Harvey, P Kilbourn, A Kucukmetin, , M Meirovitz, J Monaghan, R Rangar, N Rashid, A Richardson, B Sarker, M Sihkanyisiwe, A Tailor, G Thompson, G Wilson, B Wright, C Youlton, J Youlton.

Barts

D Oram (Lead), Usha Menon (Lead) J Ademi, C Amarasinghe, CM Baque-Juxton, S Bhola, J Bramble, J Chapman, J Charalambous, A Clough, L Cole, L Crosby, J Cunningham, E d'Tisi, E Ferrier, E Forde, P Goulding, B Heyer, J Jonsson, A Knowles, E Liu-Koo, AM Mackinson, V Medic, U Menon, A Relf, K Reynolds, B Rufford, E Ryan, S Sheik, C Stubbs, L Walters, D Warrington, J Webb.

Liverpool

J Herod (Lead), C Atherton, T Aust, L Bailey, S Bassi, L Baty, M Brown, H Burgin, J Carter, J Chapman, B Cheetham, JP Conway, H Crocker, B Daniels, L Diment, A Drought, C Finnegan, K Ford, L Greenfield, S

Hailward, J Hazelton, M Herod, S Inwood, S Jones, V Jones, L Korb, H Lee, L Limbert, K Lord, J Maloney, M Maraj, J McCarthy, L McGlynn, D Ndlela, J Newman, A Nicolson, K Pearson, S Pennington, D Petter, P Stewart, A Tannock, B Thomson, A Webster, J Webster, S West, H Wright, G Zabroski.

Nottingham
K Williamson (Lead), E Bailey, V Barker, J Barkes, C Bower-Smith, A Bowley, C Bown, S Chowdhary, C Church, V Clements, S Colbeck, F Dack, B Gibbs, M Gill, V Hessom, C Hewitt, R Hutchinson, C Hynes, J Kythreotis, L Lacy, M Mahal, K Manderson, E Mercer, C Norris, D Nunns, C Oakley, T Parkes, C Reynolds, R Rock, H Rushbrook, C Sampson, K Sihra, S Sinclair, Z Thomas, S Thompson, S Vimplis, H Ward, N Ward, K Warner, G Wilson.

Manchester
MW Seif (Lead), K Reynolds (Lead), G Atanga, S Atkinson, L Bailey, C Barber, P Bhakar, N Bhandari, A Blackman, K Bowden, S Briggs, J Brown, D Bushell, K Butler, S Charles, J Collins, M Condon, M Dale, M Doyle, J Dunscombe, R Elfin, R Elven, M Faheem-Siddiqui, MR Green, Z Griffiths, J Harris, N Harwood, J Hawnaur, H Haydock, S Heywood, P Hughes, L Ivers, S Kaye, C Kilkelly, J Lees, M Maheem, G Martin, S Mawn, S McDonald, M Moore, T Morgan, J Nelson, E Oughton, A Panteli, V Parker, J Peacock, C Philipps, J Prior, V Purnell, S Renshaw, L Roberts, S Robin, J Robinson, R Simpson, T Speakman, F Storton, S Subin, J Taylor, X Vanakara, A Webb, C Webb, C Wilde, M Williams, V Williams, A Wood, C Wood, H Wright.

Derby
H Jenkins§ (Lead), I Scott (Lead)A Bali, J Barke, C Benson, C Bower-Smith, H Bullock, J Caborn, S Crockett, A Ferguson, G Forbes, J Gomes, R Harrison, C Hollins, M Jones, A North, R Rock, , M Scott, H Stanton, S Thompson, M Tudge, G Turner, J Weston, C Williams.

Royal Free
T Mould (Lead), I Aitken, S Amin, I Beal, S Bhola, S Blackmoore, K Borroughs, H Brown, S Burke , D Colia, L Crosby, G Desai, C Efueye, H Evans, E Ferrier, K Fitzgerald, G Fletcher, C Fox, G Gaston, K Harvey, B Heyer, T Hitchen, K Isherwood, E Izard, E Koo, M Lagos, K Lakhani, AM Mackinson, L McKenzie, V Medic, S Mohamed, S More, K Muir, W Myburg, N Nayak, O Ojo, N Old, A Oldham, S Porcherot, E Rawstron, M Sharma, T Stevens, C Sundstrom, E Sweeney, J Terwin, D Townshend, F Turner, S Wellington, D White, J Wickes, L Young, E Zard.

Portsmouth
R Woolas (Lead), S Aldcock, M Anderson, E Barclay, ? Bell, R Bonner, E Bowes, D Brinkmann, J Burns, K Chorley, C Dhar, K Fairley,F Gardner, B Gibbs, Y Griffiths, R Harrison, L Hayward, C Ihezue, C Isaac, D James, F Jones, D Mason, E Merritt, R Morris, M Oakey, J Skinner, S Tilbury, J Turpitt, A Webb, C West, J Woolas.

Bristol
J Murdoch (Lead), B Anderson, F Anderson, H Andrews, E Barrow, R Brown, J Chippett, A D'Angelo, K Gale, N Hammadieh, K Henson, A Hobbs, K Horton-Fawkes, K Hunstman, T James, N Jeal, S King, E Langdon, M Lord, J Marsden-Williams, K McMillan, V Mitchell, K Nicholls, D Park, R Phillips, C Pretsell, RC Sanders, B Schaefer, C Shahin, S Shahin, S Sizer, P Taylor, M Tovey, N Vickers, S Wilmot.

Belfast
S Dobbs (Lead), M Alkalbani, M Carey, J Caruth, M Clarke, J Forbes, M Gallagher, B Gibbs, M Hendron, R McClelland, M McComskey, D Morgan, M Murray, A O'Donnell, C Poh, J Price, H Sheldon, M Yoong, A Zawislak.

Cardiff

N Amso (Lead), S Basu, D Bell, H Clarke, A Evans, J Evans, T Griffiths, P Henderson, R Howells, R Jones, G Jose, G Looker, C Morgan, G Rieck, A Rogers, A Sharma, A Sims, S Underwood, D Williams, G Williams.

North Wales
S Leeson (Lead), A Baker, V Byrne, C Chapman, B Davies, S Edwards, J Galley, L Griffiths, S Hogg, JY Houghton-Wright, M Howarth, K Hughes, M Hughes, H Jones, D Longley, A Roberts, L Sharpe, S Thomas, BJ Turner, B Warmington, B Waterson.

Middlesbrough
D Cruickshank (Lead), A Bullen, V Chadwick, K Chapman, J Francis, R Goldie, C Ikwan-McCabe, K Jan, D Khan, L Lewis, J Nevin, L Prentis, J Proll, R Shanbhag, G Tarr
